# Desiccation of the leaf mesophyll and its implications for CO_2_ diffusion and light processing

**DOI:** 10.1111/pce.14287

**Published:** 2022-03-03

**Authors:** Mina Momayyezi, Aleca M. Borsuk, Craig R. Brodersen, Matthew E. Gilbert, Guillaume Théroux‐Rancourt, Daniel A. Kluepfel, Andrew J. McElrone

**Affiliations:** ^1^ Department of Viticulture and Enology University of California Davis California USA; ^2^ School of the Environment Yale University New Haven Connecticut USA; ^3^ Department of Plant Sciences University of California Davis California USA; ^4^ Institute of Botany Universität für Bodenkultur Vienna Austria; ^5^ USDA‐ARS Crops Pathology and Genetics Research Unit Davis California USA

**Keywords:** 3D leaf complexity, drought stress, leaf carbon‐water exchange, leaf structure and function, light absorption profiles, mesophyll conductance, X‐ray microcomputed tomography

## Abstract

Leaves balance CO_2_ and radiative absorption while maintaining water transport to maximise photosynthesis. Related species with contrasting leaf anatomy can provide insights into inherent and stress‐induced links between structure and function for commonly measured leaf traits for important crops. We used two walnut species with contrasting mesophyll anatomy to evaluate these integrated exchange processes under non‐stressed and drought conditions using a combination of light microscopy, X‐ray microCT, gas exchange, hydraulic conductance, and chlorophyll distribution profiles through leaves. *Juglans regia* had thicker palisade mesophyll, higher fluorescence in the palisade, and greater low‐mesophyll porosity that were associated with greater gas‐phase diffusion (*g*_IAS_), stomatal and mesophyll (*g*_m_) conductances and carboxylation capacity. More and highly‐packed mesophyll cells and bundle sheath extensions (BSEs) in *Juglans microcarpa* led to higher fluorescence in the spongy and in proximity to the BSEs. Both species exhibited drought‐induced reductions in mesophyll cell volume, yet the associated increases in porosity and *g*_IAS_ were obscured by declines in biochemical activity that decreased *g*_m_. Inherent differences in leaf anatomy between the species were linked to differences in gas exchange, light absorption and photosynthetic capacity, and drought‐induced changes in leaf structure impacted performance via imposing species‐specific limitations to light absorption, gas exchange and hydraulics.

## Summary Statement

Species‐specific differences in leaf anatomy between *Juglans regia* and *Juglans microcarpa* were linked to differences in CO_2_ diffusion, light absorption, water transport and carboxylation capacity. Drought‐induced changes in leaf structure impacted performance via CO_2_, water and light absorption.

## 1 INTRODUCTION

Photosynthesis supports plant growth, development, and reproduction, and to optimise this process, leaves must balance light absorption, carbon capture, and water loss under ever changing conditions. Anatomical and physiological leaf traits play key roles in determining the exchange of light, CO_2_, and water with the environment. How the structural and physiological components of the leaf are affected by drought is of increasing importance given the increasing frequency and duration of drought globally (Brodribb et al., 2020; Choat et al., 2018). Stomata regulate the diffusion of gases across the leaf surface, where water vapour is lost in exchange for CO_2_ after crossing the leaf boundary layer, and respond strongly to changes in vapour pressure and soil moisture in many species to minimise water loss (Cowan & Troughton, 1971; Farquhar & Sharkey, 1982; Mott & Peak, 2013; Oren et al., 1999; Turner et al., 1984). After reaching the substomatal cavity, CO_2_ molecules are subject to a series of gas and liquid phase resistances along the diffusion pathway through the intercellular airspace, cell walls, membranes, cytosol, and other cellular components to reach carboxylation sites inside chloroplasts. The inverse of the sum of these resistances is used to calculate mesophyll conductance (*g*_m_, see Table 1 for symbol definitions), (Flexas et al., 2008; Flexas et al., 2018; Tosens & Laanisto, 2018), and these resistances to the movement of CO_2_ should be sensitive to changes in leaf water status. Theoretical predictions and experimental observations have found that both the physical properties of the mesophyll (e.g., cell wall thickness, palisade and spongy mesophyll cell density, mesophyll surface area exposed to the intercellular airspace—IAS) and the underlying physiology (i.e., chloroplast positioning, aquaporins, and carbonic anhydrase activity) strongly influence CO_2_ diffusion within a leaf and its concentration at the sites of carboxylation (Flexas et al., 2012; Momayyezi & Guy, 2017a, 2017b, 2018; Muir et al., 2014; Théroux‐Rancourt & Gilbert, 2017; Tholen & Zhu, 2011). The products of photosynthesis are then either consumed locally or exported to the vascular tissue.

**Table 1:** List of traits and variables used

Variable	Definition	Unit
*A*_n_	Net assimilation rate	µmol CO_2_ m^−2^ s^−1^
*A*_max_	Maximum assimilation rate at saturating CO_2_	µmol CO_2_ m^−2^ s^−1^
BSEs	Bundle sheath extensions	Dimensionless
*C*_i_	Intercellular airspace CO_2_ concentration	µmol mol^−1^
*C*_i_*	Intercellular CO_2_ photocompensation point	µmol mol^−1^
*C*_c_	Chloroplast CO_2_ concentration	µmol mol^−1^
*E*	Transpiration rate	mmol m^−2^ s^−1^
*g*_IAS_	Intercellular airspace (gas phase) conductance	mol m^−2^ s^−1^ bar^−1^
*g*_liq_	Liquid phase conductance	mol m^−2^ s^−1^ bar^−1^
*g*_m_	Mesophyll conductance	mol CO_2_ m^−2^ s^−1^
*g*_s_	Stomatal conductance	mol m^−2^ s^−1^
*g*_smax_	Maximum stomatal conductance	mol m^−2^ s^−1^
*K*_leaflet_	Leaflet hydraulic conductance	mmol m^−2^ s^−1^ MPa^−1^
*L*_leaf_	Leaf thickness	μm
*L*_mes_	Mesophyll thickness	μm
*L*_epi‐adaxial_	Adaxial epidermis thickness	μm
*L*_epi‐abaxial_	Abaxial epidermis thickness	μm
PPFD	Photosynthetic photon flux density	µmol m^−2^ s^−1^
*SA*_mes_/*V*_mes_	Mesophyll surface area exposed to the intercellular airspace per mesophyll volume	μm^2^ μm^−3^
*V*_IAS_/*V*_mes‐cell_	Intercellular airspace volume to mesophyll cell volume	m^3^ m^−3^
*WUE*_i_	Intrinsic water use efficiency	µmol CO_2_ mol^−1^ H_2_O
Ψ_leaflet_	Leaflet water potential	MPa
Φ_PSII_	Quantum yield of photosystem II	Dimensionless
Γ*	Chloroplast CO_2_ photocompensation point	µmol mol^−1^
*R*_d_	Dark respiration	µmol m^−2^ s^−1^
θ_IAS_	Mesophyll porosity	m^3^ m^−3^
τ_leaf_	Tortuosity	m^2^ m^−2^
*λ*_leaf_	Lateral path lengthening	m m^−1^

Similarly, but in an opposing flow direction, water exits the vascular tissue and travels through the mesophyll, ultimately evaporating into the IAS and lost to the atmosphere via the stomata or across the epidermis. A primary role of the leaf vasculature is therefore to replace the water lost while the stomata are open to sustain the uptake of CO_2_ for photosynthesis. During drought, insufficient soil moisture or declines in the hydraulic conductance of the vascular system fail to meet the evaporative demands of leaves, leading to loss of turgor in the mesophyll. Recent work has shown that turgor loss directly influences cell shape and leaf porosity even while the xylem remains functional (Scoffoni et al., 2017), and the resulting structural and physiological changes associated with leaf desiccation can significantly alter leaf hydraulic conductance (Buckley et al., 2017; Scoffoni et al., 2014), highlighting the complex sequence of events that take place inside the leaf during drought.

An additional layer of complexity can be observed in the overall structure of the leaf mesophyll and the embedded vasculature, which should not only be organised to facilitate the movement of both carbon and water, but also optimised for the opposing gradients of light and CO_2_ within the leaf (Borsuk & Brodersen, 2019; Evans, 1999; Evans, 2021; Smith et al., 1997; Xiao et al., 2016). A general assumption is that the absorptive, optical, and hydraulic properties of leaves are optimised under well hydrated conditions with the mesophyll cells under full turgor. What then are the effects of turgor loss and the associated changes in cell shape and volume on the processing of light, CO_2_ and water as leaves dehydrate? As mesophyll cells lose turgor, there should be consequences for the different physiological roles that those cells contribute to, and perhaps differently in the palisade and spongy mesophyll based on cell size and shape. For example, loss of turgor in the mesophyll should lead to changes in the physical shape of the cells (Canny et al., 2012), which has implications for the surface area exposed to the IAS, the tortuosity of the diffusion pathway for both H_2_O and CO_2_ (i.e., decline in *g*_m_, Cano et al., 2014) by bringing the epidermis closer to the sites of evaporation within the leaf (Buckley et al., 2017). However, it should also directly affect the optical properties of those cells for light propagation and scattering, leading to sub‐optimal light absorption with negative impacts on biochemical activity and light use during photosynthesis. The distribution of mesophyll cells and presence of bundle sheath extensions (BSEs) can influence light distribution with depth into a leaf (Evans & Vogelmann, 2003; Holloway‐Phillips, 2019; Smith et al., 1997). Numerous studies have shown significant relationships between optical properties (e.g., absorptance and reflectance) and leaf chlorophyll concentration under water stress (e.g., Carter, 1993; Carter & Knapp, 2001; Gitelson et al., 2003), however, the functional relationship between mesophyll and light absorption with depth into a leaf and under dehydration is not known. Recent studies evaluating mesophyll anatomy at finer scales have shown links between biophysical properties of mesophyll cells and IAS conductance (*g*_IAS_). These linkages are associated with variation in airspace tortuosity (i.e., the ratio of the diffusive path length to the straight path length; τ_leaf_), porosity (i.e., IAS volume fraction of the mesophyll; θ_IAS_), and path lengthening as a consequence of CO_2_ diffusion through each distinct stomate to IAS (λ_leaf_) (Earles et al., 2019; Gommes et al., 2009; Harwood et al., 2021; Théroux‐Rancourt et al., 2021; Tosens et al., 2016). Little is known about how water stress influences these relationships, as suggested in CO_2_ and water flux models considering mesophyll and vascular geometry (Rockwell et al., 2014c, 2017).

Declines in the net assimilation rate (*A*_n_) under water stress are well documented, and arise due to both stomatal and non‐stomatal limitations. Loss of turgor in the guard cell complex creates a physical barrier for the diffusion of CO_2_ into the leaf, and leads to a depletion of the internal CO_2_ supply to carboxylation sites, but also negatively influences photochemistry due to increases in leaf temperature (Brodribb & Holbrook, 2003; Buckley, 2019; Buckley et al., 2017; Galle et al., 2009). Excessive leaf temperatures and desiccation can also lead to permanent damage to photosynthetic machinery (Cano et al., 2013; Chaves et al., 2009; Galmés et al., 2007; Hsiao, 1973; Nadal & Flexas, 2018; Trueba et al., 2019; Urban et al., 2017). A negative response of *g*_m_ to dehydration occurs under mild water stress, and this response is exacerbated by high light intensity (Flexas et al., 2008; Galle et al., 2009; Zhou et al., 2007), illustrating, the need to understand the coordination of multiple exchange processes since excess light can be detrimental to the photosynthetic machinery when rates of carbon fixation decrease with water stress.

The goal of this study is to unfold the complex links between the leaf anatomical traits and functional diversity in CO_2_, water and light absorption. Here, we explore inherent differences in leaf structure for two walnut species with leaf anatomy contrasting in the fraction of BSEs in relation to functional responses under non‐stressed condition and impacts of stress‐induced changes in leaf anatomy on species performance, and tested several hypotheses based on our preliminary observations for these species. We used *Juglans regia* L., native to central Asia, Himalayas, China and southeastern Europe (McGranahan & Leslie, 2009) and *J. microcarpa* Berland. var. *microcarpa*, native to southwestern United States and northwestern Mexico, which are adapted to contrasting environments with different water and light availabilities (McGranahan & Leslie, 2009). Our preliminary greenhouse and field measurements indicate differences between species in gas exchange capacity and leaf anatomy, with *J. microcarpa* showing a higher fraction of BSEs within the leaf. We expected that inherent differences in BSEs and mesophyll cell packing will affect light absorption profiles and CO_2_ diffusion in two walnut species. *Juglans regia*, with elongated and densely stacked palisade mesophyll and more porous lower mesophyll was hypothesised to show higher upper‐mesophyll light absorption (Cui et al., 1991), and greater intercellular airspace diffusion. Previous studies reported species with more BSEs have greater structural rigidity and lower turgor loss point and show less shrinkage in leaf and mesophyll cells under dehydration (Pivovaroff et al., 2014, Scoffoni et al., 2017). Therefore, we expected *J. regia* leaves with less structural and functional support by BSEs (mainly known as parenchyma cells connecting veins to epidermis) to exhibit more volumetric changes through mesophyll cells, porosity, and *g*_IAS_ under dehydration. In contrast, *J. microcarpa* with higher cell packing and BSEs was expected to more reflect small changes in cell geometry through light absorption profile, as suggested in species with dense spongy mesophyll through more lower‐mesophyll scattering impact (Ren et al., 2019; Smith et al., 2004). To evaluate these hypotheses, we used X‐ray micro‐computed tomography (microCT) imaging to observe in‐depth variation in leaf and cell morphology with dehydration coupled with gas exchange measurements.

## 2 MATERIALS AND METHODS

### 2.1 Plant materials and growth conditions

*Juglans regia* cv. Chandler is the most common hybrid scion from natural populations of *J. regia* L., and *J. microcarpa*, is used in *J. microcarpa* × *J. regia* crosses to produce rootstocks with resistance to crown gall and root rot diseases (Browne et al., 2015; Hasey, 2016; McGranahan & Leslie, 2009). *Juglans microcarpa* is reported to be more tolerant to water deficit (Knipfer et al., 2020).

Two‐year‐old saplings of clonal and non‐grafted *J. regia* and *J. microcarpa* were grown under consistent greenhouse light and temperature condition, and shipped from the University of California, Davis to the Marsh Botanical Garden greenhouse at Yale University, and were allowed to acclimate under well‐watered conditions (without any pre‐drought hardening) for 4 weeks before use in the experiments. The gradual dry down procedure was done by reducing water application to 75% of full‐irrigation during the first week and then reducing it further to 50% of full‐irrigation in the second week of drying. Eight saplings for each species were randomly assigned to either a well‐watered control treatment (200 ml water per day) or a water stress treatment with 50% less water than controls (100 ml water per day), equal to daily water loss from pots under each treatment. This watering regime was then maintained until the completion of the experiment. Using the method as described by Knipfer et al. (2020), water loss through transpiration and water evaporation from the soil were quantified during the experiment to calculate the required amount of water under each treatment.

During growth and experimental stages, plants were under supplemental lighting (PPFD = 500 µmol m^−2^ s^−1^) with a 16‐h photoperiod, maximum temperature of 25°C during day and minimum of 18°C during night in the greenhouse, in 2.65‐L pots containing a 40% pine bark, 40% sphagnum peat moss and 20% vermiculite. The two irrigation treatments were maintained for approximately 2 weeks before the measurements.

### 2.2 Photosynthesis measurements

Net assimilation rate (*A*_n_), stomatal conductance (*g*_s_) and the intercellular airspace CO_2_ concentration (*C*_i_) were measured on the 4th or 5th leaflet of the most recent fully expanded leaf using LI‐COR 6400 XT and LI‐COR 6800 systems fitted with 6400‐40 and 6800‐01A fluorometers, respectively (see Supporting Information Method for *A‐C*_i_ and *A‐I* curves). All measurements were done under PPFD = 1500 (10% blue vs. 90% red) (µmol m^−2^ s^−1^), chamber temperature at 25°C, ambient chamber CO_2_ concentration (*C*_a_) at 400 (µmol mol^−1^), flow rate at 150 (µmol air s^−1^), and vapour pressure deficit between 1.5 and 2.0 kPa. All leaflets were dark adapted for 20 min before all other measurements to obtain the maximum quantum yield of photosystem II. The quantum yield of photosystem II (Φ_PSII_) under actinic light was obtained by application of saturating multiphase flashes (>8000 µmol m^−2^ s^−1^) as per Genty et al. (1989).

### 2.3 Stable carbon isotope discrimination method

Pre‐evacuated 10 ml gas tight vials (Exetainer, Labco, UK) were used to collect air exiting the LI‐COR chamber through a tube connected to the cuvette exhaust, either with (CO_2_P = plant CO_2_) or without (CO_2_R = reference CO_2_) leaf material inside the chamber. The air exiting the LI‐COR cuvette was collected as described by Théroux‐Rancourt and Gilbert (2017) and analysed for stable carbon isotope composition. A three‐way valve was added to the LI‐COR 6800 chamber through the exhaust tube. A ~2 m sampling tube was connected to the third port, and the valve was opened towards it. After ~5 min, the valve was returned to its primary position along the chamber exhaust tube, and 15 ml air was collected from the tube into a gas‐tight glass syringe through a brass luer‐lock fitting. A needle was connected to the syringe, the syringe's valve was opened, and 3 ml of air sample was flushed through the needle before purging 12 ml of the air into a vial. Sampling started with CO_2_R samples, followed by CO_2_P and then alternating CO_2_R with one CO_2_P sample. After taking the first CO_2_R sample, a leaf was placed inside the chamber and light adapted for 20 min before taking the first CO_2_P sample. The same protocol was followed for every plant sample, ending with a final CO_2_R sample. Gas exchange and chlorophyll fluorescence measurements were recorded during each sampling for CO_2_P.

Vials were transferred to the Stable Isotope Facility, at the University of California Davis within a week for measuring carbon isotope discrimination on ThermoScientific GasBench system II interfaced to a ThermoScientific Delta V Plus isotope ratio mass spectrometer (ThermoScientific). Through a six‐port rotary valve (Valco), CO_2_ was sampled using a 250 μl loop programmed to switch at the maximum CO_2_ concentration in the helium carrier gas. N_2_O and other gases were trapped and separated from CO_2_ by moving through a PoraPLOT Q column (25 m × 0.32 mm ID, 2.5 ml min^−1^) set at 50°C at the mass spectrometer. A pure CO_2_ standard tank of 400 µmol mol^−1^ was used to calculate provisional *δ* values of samples. The system was referenced against internal laboratory standards which were calibrated against NIST 8545 isotopic standards to correct provisional *δ* values. Final δ^13^C values were corrected and expressed relative to the international Vienna PeeDee Belemnite standard.

### 2.4 Calculation of *g*_m_ from carbon isotope discrimination

Ribulose‐1,5‐bisphosphate carboxylase/oxygenase (Rubisco) discriminates against ^13^CO_2_ relative to ^12^CO_2_ during carboxylation (Guy et al., 1993). The amount of discrimination expressed in vivo depends on the diffusion gradient for CO_2_ from the bulk atmosphere. By comparing the observed discrimination (${∆}_{o}$) with the predicted discrimination (Δ_i_) based only on the diffusion gradient through the stomata (i.e., *C*_a_ to *C*_i_), the gradient associated with the remaining portion of the diffusion pathway (i.e., *C*_i_ to *C*_c_) can be estimated and used to calculate *g*_m_ (Evans et al., 1986). Smaller contributions to total discrimination, associated with respiratory (Δ_e_) and photorespiratory carbon flux (Δ_f_), must also be accounted for. The effect of *g*_m_ on overall isotope discrimination (Δ_gm_) is then given by:

(1) $$\Delta_{g_{m}}=\Delta_{i}-\Delta_{o}-\Delta_{e}-\Delta_{f}$$

Observed discrimination was calculated according to Evans et al. (1986):

(2) $${∆}_{o}=\frac{1000\zeta \left(\delta^{13}C_{a}-\delta^{13}C_{e}\right)}{1000+\delta^{13}C_{a}-\zeta \left(\delta^{13}C_{a}-\delta^{13}C_{e}\right)}$$

(3) $$\zeta =\frac{C_{e}}{\left(C_{e}-C_{a}\right)}$$

where, $\delta^{13}C_{e}\;\text{and}\;\delta^{13}C_{a}$ are the isotopic ratios of reference CO_2_ and unconsumed CO_2_, respectively. ζ is the ratio of the reference CO_2_ concentration (*C*_e_) entering the cuvette, as determined by the LI‐COR 6800, and the net amount consumed in photosynthesis (i.e., *C*_e_ – *C*_a_).

Predicted discrimination was calculated from gas exchange data with corrections for ternary effects as per Farquhar and Cernusak (2012):

(4) $${∆}_{i}=\frac{1}{\left(1-t\right)}a\prime +\frac{1}{\left(1-t\right)}\left(\left(1+t\right)b-a\prime \right)\frac{C_{i}}{C_{a}}$$

where *b* is the fractionation in carboxylation of ribulose bisphosphate catalysed by Rubisco (−29‰; Guy et al., 1993). The ternary correction factor, *t*, is:

(5) $$t=\frac{\left(1+a\prime \right)E}{2g_{\mathrm{ac}}^{t}}$$

where *E* is the transpiration rate and $g_{\mathrm{ac}}^{t}$ is the combination of boundary layer and stomatal conductance to CO_2_. The combined factor for diffusional fractionation through stomata and the boundary layer, a′, is:

(6) $$a\prime =\frac{a_{b}\left(C_{a}-C_{s}\right)+a\left(C_{s}-C_{i}\right)}{\left(C_{a}-C_{i}\right)}$$

where *a* and *a*_b_ are the fractionations occurring during diffusion across the stomata (4.4‰) and through the boundary layer (2.9‰), respectively, and *C*_s_ is the CO_2_ concentration at the leaf surface (Evans et al., 1986).

Discriminations associated with respiration (Δ_e_) and with photorespiration (Δ_f_) were calculated from Equations (9) and (10) (Farquhar & Cernusak, 2012):

(7) $${∆}_{e}=\frac{1+t}{1-t}\left[\frac{{\mathrm{eR}}_{d}}{\left(A_{n}+R_{d}\right)C_{a}}\right]\left(C_{i}-\Gamma *\right)$$

(8) $${∆}_{f}=\frac{1+t}{1-t}\left[ƒ\frac{\Gamma *}{C_{a}}\right]$$

where *e* and *ƒ* are the fractionations associated with respiration and photorespiration, respectively. We assumed *ƒ* to be −11.6‰ (Lanigan et al., 2008) and that there is no significant fractionation associated with dark respiration during the day (Wingate et al., 2007). However, because respired carbon was likely fixed during prior photosynthesis in the greenhouse, we took *e* to equal the difference between δ^13^ *C*_e_ (−32 to −37‰) and the isotopic composition for atmospheric CO_2_ (δ^13^ *C*_atm_) in the greenhouse (assumed to be −8‰; Alonso‐Cantabrana & von Caemmerer, 2015):

(9) $$e=\delta^{13}C_{e}-\delta^{13}C_{\mathrm{atm}}$$

Discrimination associated with *g*_m_ is described by Farquhar and Cernusak (2012):

(10) $$\Delta_{g_{m}}=\frac{1+t}{1-t}\left[b-a_{i}-\frac{eR_{d}}{A+R_{d}}\right]\frac{A_{n}}{g_{m}C_{a}}$$

where *a*_i_ is the fractionation factor associated with hydration and diffusion in water (1.8‰ at 25°C). Substituting Equation (3) into Equation (12) and rearranging, *g*_m_ was then calculated as:

(11) $$g_{m}=\frac{1+t}{1-t}\left[b-a_{i}-\frac{eR_{d}}{\left(A_{n}+R_{d}\right)}\right]\frac{A_{n}}{C_{a}}/\left({∆}_{i}-{∆}_{o}-{∆}_{e}-{∆}_{f}\right)$$

### 2.5 Calculation of *C*_c_

Having obtained *g*_m_ by the chlorophyll fluorescence method, the CO_2_ concentration in the chloroplast (*C*_c_) was estimated:

(12) $$C_{c}=C_{i}-\frac{A_{n}}{g_{m}}$$

*g*_m_ obtained from the stable isotope discrimination method was strongly correlated with that estimated using the chlorophyll fluorescence method (see Supplementary Methods) (*g*_m_ values between 0.03 and 0.19; *R* ^2^ = 0.8016, *p* < 0.0001; Figure S1). Given the potential uncertainties with *g*_m_ estimates obtained from the variable *J* method, and the increased sensitivity of certain methods for leaves experiencing water stress, we chose to present *g*_m_ from data carbon isotopic discrimination technique.

### 2.6 *A*_n_‐*C*_i_ and *A*_n_‐*I* curves

To better understand photosynthetic responses under dehydration, we constructed CO_2_ (*A*_n_‐*C*_i_) and light response (*A*_n_‐PPFD) curves for each species. *A*_n_‐*C*_i_ curves were constructed for all individuals at 1500 µmol m^−2^ s^−1^ PPFD under the following sample CO_2_ concentration: 400, 50, 80, 100, 150, 200, 400, 600, 800, 1000, 1200, 1500 ppm. Leaflets from all individuals were illuminated at adaxial and abaxial surfaces, respectively at 0, 50, 100, 400, 800, 1000, 1500 µmol m^−2^ s^−1^ to measure *A*_n_‐PPFD curves at 400 µmol mol^−1^ sample CO_2_ (Figure S2).

### 2.7 Leaflet water potential measurements

The two leaflets opposite the one used for gas exchange measurements were used to measure water potentials. The first leaflet was cut at petiolule base and bagged (in a clear bag) for 10 min to allow equilibration within the leaflet. Then, using a razor blade ~1 cm of leaflet lamina was cut from either side of the middle vein to fit the short petiolule inside the pressure chamber gasket. Chamber pressure was increased slowly until the balancing pressure was reached. The second leaflet was covered in a dark bag for 20 min before removal to obtain the water potential of the rachis for the remainder of the leaf.

### 2.8 Leaflet water potential and leaflet hydraulic conductance

Leaflet water potential (Ψ_leaflet_) was measured using a pressure chamber (PMS Instrument Company, Model 1505D) immediately after gas exchange measurements between 10 a.m. to 3 p.m. (Williams & Araujo, 2002) (see Supporting Information Method).

Leaflet hydraulic conductance (*K*_leaflet_) was calculated using in situ evaporative flux method according to Brodribb and Holbrook (2003) and Simonin et al. (2015):

(13) $$K_{\text{leaflet}}=E/\Delta \Psi_{\text{bagged}\;\text{leaflet}‐\text{unbagged}\;\text{leaflet}}$$

*E* is the transpiration rate (mmol m^−2^ s^−1^) measured using gas exchange system, and ΔΨ_bagged leaflet–unbagged leaflet_ is the difference between bagged leaflet and unbagged leaflet water potential (MPa). Average unbagged and bagged Ψ _leaflet_ were −0.8 (±0.04) and −0.7 (±0.03) MPa for *J. regia* and −1.0 (±0.06) and −0.6 (±0.02) and *J. microcarpa* under well‐watered, respectively, and −1.4 (±0.09) and −1.25 (±0.06) MPa for *J. regia* and −1.7 (±0.06) and −1.4 (±0.1) MPa for *J. microcarpa* under dehydration, respectively. The Ψ_leaflet_ showed 3%–6% variability between the leaflets and the average Ψ_leaflet_ was 3%–5% more negative than Ψ_leaf_ in each species. The average transpiration rate (*E*) within and between leaflets on the same leaf were compared for these measurements. for *J. regia* and *J. microcarpa* under well‐watered (0.8 ± 0.02 and 0.7 ± 0.01 mmol m^−2^ s^−1^), and drought conditions (0.6 ± 0.03 and 0.4 ± 0.03 mmol m^−2^ s^−1^), varied by 5%–10% between leaflets. Minimal or no significant difference in *E* existed across individual leaflets for scaling to the total leaflet area.

To quantify the stomatal aperture under well‐watered and dehydrated conditions in each species, both hypostomatous, abaxial epidermis imprints using transparent nail polish (water‐based) were obtained from the same leaflets used for the gas exchange measurements. Using the imprints, stomata images were taken on a light microscope at 20x (Nikon C2^+^, Nikon Instruments Inc.) and used to measure the stomatal pore dimensions. The inner pore width (μm) was divided by the inner pore length (μm) to calculate the stomatal aperture ratio (Rui & Anderson, 2016). Maximum *g*_s_ (*g*_smax_) was calculated using the stomata pore dimensions (Franks & Beerling, 2009) and used to interpret changes in stomata opening and *g*_s_ in the two species and further, test the precision of the stomatal aperture quantification method. To calculate stomata size, guard cells length was multiplied by total width, for closed guard cells (Franks & Beerling, 2009).

### 2.9 X‐ray micro computed tomography imaging and segmentation

Intact plants with their soil were sent back to the UC Davis greenhouse and potted again where water potentials and soil water content were monitored and maintained for several days until scanning them 7 days after shipping in Lawrence Berkeley National Laboratory (LBNL) Advanced Light Source (ALS). The same leaflet samples used for gas exchange at Yale were kept intact, collected, bagged and placed in a cooler at room temperature an hour before scanning in ALS. A section of the leaflet lamina from each plant was enclosed between two pieces of Kapton tape to prevent desiccation of the tissue and sample movement during the scanning. Samples were placed inside the end of a pipette tip and scanned under a continuous tomography mode at 23 keV using 10× objective lens (pixel resolution of 0.65 μm). Raw tomographic data were reconstructed using TomoPy (Gürsoy et al., 2014) through both gridrec and phase retrieval reconstruction methods (Figure S3) (Davis et al., 1995; Dowd et al., 1999).

Five hundred consecutive slices from the grid and phase stacks were selected for segmentation. The resulting image stack was segmented using the methods presented in Théroux‐Rancourt et al. (2020) (Figure S3). Six slices were labelled manually per scan and were used to train a random‐forest model for automated segmentation of the whole scan image stack. The final segmented stacks had individual labels for the adaxial epidermis, abaxial epidermis, mesophyll cells, intercellular airspace, BSEs, veins, and background outside of the scanned leaf. This final stack was used to extract leaf anatomical traits, that is surface areas, volumes, and lengths.

### 2.10 Mesophyll surface area and porosity

**Figure 1:**
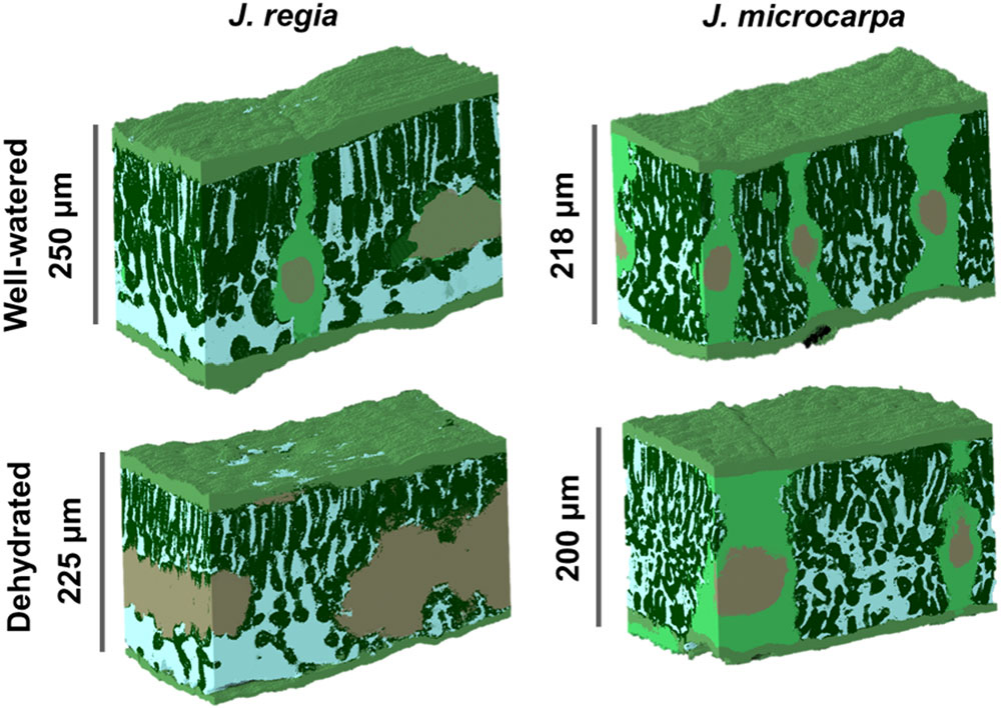
Leaf three‐dimensional projection for *Juglans regia* and *Juglans microcarpa* under well‐watered and dehydrated conditions [Color figure can be viewed at wileyonlinelibrary.com]

As described by Théroux‐Rancourt et al. (2017), mesophyll porosity, θ_IAS_ (m^3^ m^−3^) was calculated as the IAS volume as a fraction of the total mesophyll volume. The IAS volume (*V*_IAS_) to mesophyll cell volume (*V*_mes‐cell_) ratio and the mesophyll surface area exposed to the IAS (*SA*_mes_) per mesophyll volume (*V*_mes_) were calculated as *V*_IAS_/*V*_mes‐cell_ (m^3^ m^−3^) and *SA*_mes_/*V*_mes_ (μm^2^ μm^−3^), respectively (Figure 1).

### 2.11 Tortuosity and lateral path lengthening

The tortuosity factor, *τ*(m^2^ m^−2^), was defined as the ratio of the diffusive path length within the IAS to the straightest path length in the absence of any physical obstacles to diffusion between a stomate and the cell surface:

(14) $$\tau ={\left(\frac{L_{\mathrm{geo}}}{L_{\mathrm{Euc}}}\right)}^{2}$$

where geodesic distance (*L*_geo_) is the distance from the stoma to a cell surface, and Euclidean distance, (*L*_Euc_) (Earles et al., 2018). The *L*_geo_ and *L*_Euc_ were mapped and quantified for all voxels along the mesophyll surface and *τ* was calculated for the whole 3D image array as in Earles et al. (2018). Then, leaf‐level tortuosity (*τ*_leaf_) was calculated as the mean of *τ* values at the edge of mesophyll cells. The lateral path lengthening, *λ* (m m^−1^) was calculated using *L*_Euc_, and a second distance map as described by Earles et al. (2018) to measure the shortest unobstructed distance in a straight line between the abaxial epidermis and all points along the mesophyll surface, *L*_epi_ (Legland et al., 2016):

(15) $$\lambda =\frac{L_{\mathrm{Euc}}}{L_{\mathrm{epi}}}$$

Similarly, leaf‐level lateral path lengthening (*λ*_leaf_), was then calculated as the mean of *λ* values at the edge of mesophyll cells.

### 2.12 IAS conductance

The *τ*_leaf_, *λ*_leaf_ and $\theta_{\mathrm{IAS}}$ were used to calculate leaf‐level IAS conductance (*g*_IAS_), where *D*_m_ is the diffusivity of CO_2_ in air (m^2^ s^−1^). Diffusion path length in gas phase was equal to half of the mesophyll thickness (*L*_mes_) for hypostomatous leaves (Earles et al., 2018; Niinemets & Reichstein, 2003; Tomás et al., 2013):

(16) $$g_{\mathrm{IAS}}=\frac{\theta_{\mathrm{IAS}}D_{m}}{0.5L_{\mathrm{mes}}\tau_{\mathrm{leaf}}\lambda_{\mathrm{leaf}}}$$

### 2.13 Porosity profiles

**Figure 2:**
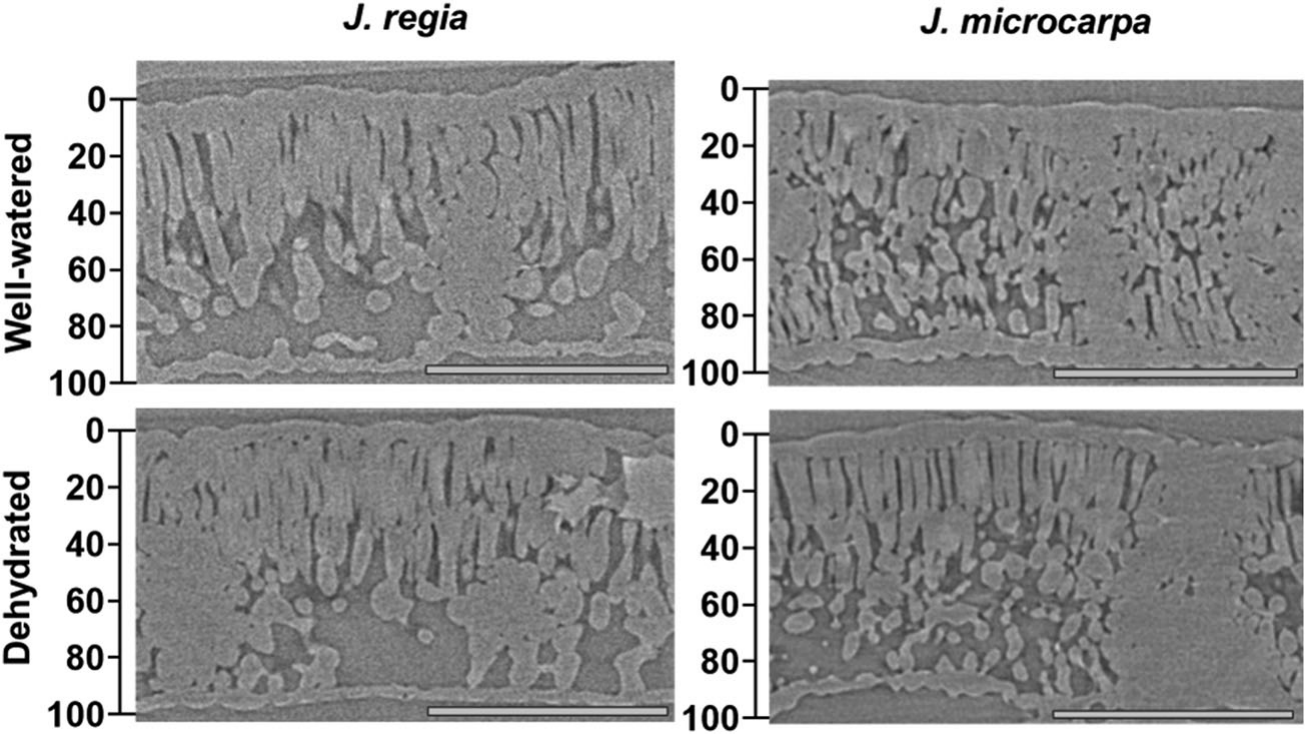
MicroCT images, representative slices from scans of *Juglans regia* and *Juglans microcarpa* leaves under well‐watered and dehydrated conditions. Bar equals 250 μm

MicroCT scans for each species under well‐watered and dehydrated conditions (Figure 2) were used to determine porosity profiles from IAS distribution with leaf depth using a plot profile of grey value distribution across leaf excluding adaxial and abaxial epidermis tissue. The grey values were used to calculate air volume for a known mesophyll area (4 μm^2^) and based on mesophyll thickness (μm) per individual within each depth after converting pixel to distance (pixel resolution of 0.65 μm).

### 2.14 Palisade mesophyll cell diameter at paradermal section

The grid reconstructions of microCT images were used to compare the palisade mesophyll cell diameter through paradermal sections at three depths (20%, 40% and 60%) from the adaxial surface in well‐watered versus dehydrated leaves within 0.02 mm^2^ of the mesophyll area.

### 2.15 Relative chlorophyll distribution through the leaf profile

We used previously reported methods to obtain chlorophyll distribution (Borsuk & Brodersen, 2019; Vogelmann & Evans, 2002) and light absorption profiles (Brodersen & Vogelmann, 2010; Koizumi et al., 1998; Takahashi et al., 1994; Vogelmann & Evans, 2002; Vogelmann & Han, 2000) for each species (Figure S4). Chlorophyll distributions were obtained by calculating the relative chlorophyll fluorescence (proportional to chlorophyll concentration) at each relative depth. Light absorption gradients, representing relative chlorophyll distribution patterns were measured using chlorophyll fluorescence imaging of leaf cross sections under direct illumination (Vogelmann & Evans, 2002; Vogelmann & Han, 2000). Fresh samples in a subset of three were cut into ~1 cm^2^ from the same leaflets and placed on top of a wet paper to protect the specimen from desiccation in a glass holder on the microscope stage (Olympus BX60, Olympus America Inc., Center Valley, PA, USA). The sample was irradiated by a broad‐spectrum LED light source at cross‐sectional direction (epi‐illumination at 490 nm; beam radius ~1 mm) (Figure S5). For adaxial or abaxial profile imaging, leaves were irradiated with direct light in sequence with monochromatic red (660 nm), green (532 nm), or blue (488 nm) light obtained from three lasers one at a time (laser spot radius = 1 mm; red solid state laser: Model #BWN‐660–10E, BandW Tek Inc.; green solid state laser: Model # DY20B, Power Technology Inc.; and blue argon gas laser: Model # Innova 300, Coherent Inc.). Using a digital Peltier‐cooled CCD camera (PIXIS 1024B, Princeton Instruments, Trenton, NJ, USA) with shutter times of 70–150 ms, emitted light of chlorophyll fluorescence was imaged after passing through a barrier filter (680 nm, half band width = 16 nm, S10–680F; Corion Filters). Light intensity through the leaf was measured in Image J (Rueden et al., 2017) from the adaxial edge of the mesophyll to the abaxial edge of the mesophyll using the line profile tool averaged over a width of 50–100 pixels (100‐pixel width was equivalent to ~60 μm at 20x magnification or ~120 μm at 10x magnification) and excluded conspicuous non‐photosynthetic structures such as epidermal cells and veins. The obtained values per each profile were normalised by dividing them by the chlorophyll fluorescence depth maxima. An absolute fluorescence intensity could not be estimated, first, due to lack of flexibility in accounting variation in light exposure needed for different samples sizes with different focal points, and second, the overall decline in fluorescence intensity under a continuous supply of light over time, that is, temporal variation in detected fluorescence signal due to Kautsky decay (Borsuk & Brodersen, 2019; Vogelmann & Han, 2000).

### 2.16 Bundle sheath extensions area

MicroCT scans for each species were used to calculate the ratio of the BSEs area where parenchyma cells connecting vascular tissue to both epidermises, as a percentage from the mesophyll cross sectional area (i.e., area of mesophyll cells and airspace) using Image J (Griffiths et al., 2013).

(17) %BSEs area=BSEs area/(BSEs area+mesophyll area)

Green light penetrates deeper into leaf and is absorbed more equally throughout the leaf profile (Brodersen & Vogelmann, 2010). Therefore, adaxial fluorescence images from green laser illumination, as described above, were used to determine the relative fluorescence for mesophyll tissue adjacent to BSEs and plot their distribution along the mesophyll in Image J. Fluorescence percentage near BSEs were normalised using maximum values per sample and plotted using epi‐ and adaxial illumination imaging data for the two species.

### 2.17 Statistics

Linear regression lines were used to describe relationships between *A*_n_ and *C*_i_ and paired *t*‐tests were used to compare differences in estimated *g*_m_ from the isotope discrimination and chlorophyll fluorescence methods using GraphPad prism 8 (GraphPad Software, Inc). Mixed linear models were used to compare treatments effects on the following physiological variables: *A*_n_, *g*_m_, *g*_s_, *C*_i_, *C*_c_, Φ_PSII_, Ψ_leaflet_, τ_leaf_, θ_IAS_, *L*_leaf_, *L*_mes_, *L*_epi‐adaxial_, *L*_epi‐abaxial_, *SA*_mes_/*V*_mes_, *V*_IAS_/*V*_mes‐cell_, *λ*_leaf_ and *g*_IAS_ in the two species under well‐watered and dehydrated treatments using SAS 9.4 (SAS Institute Inc). Adjusted *p*‐value (=0.0083) was calculated by dividing α (=0.05) by number of mean pairs per test (*n* = 6). Mixed linear models were used to compare absolute and percentage reductions for all the physiological variables relative to the well‐watered (*p* = 0.05). Number of vein emboli (see results for method description), BSEs area, and palisade diameter were compared using mixed linear models (*p* = 0.05). Logarithm or squared transformations were performed to meet normality and equal variance assumptions where needed. Multiple *t*‐tests were used for a pairwise comparison between all pairs of means (*p* = 0.05).

## 3 RESULTS

### 3.1 Mesophyll traits and IAS parameters

**Figure 3:**
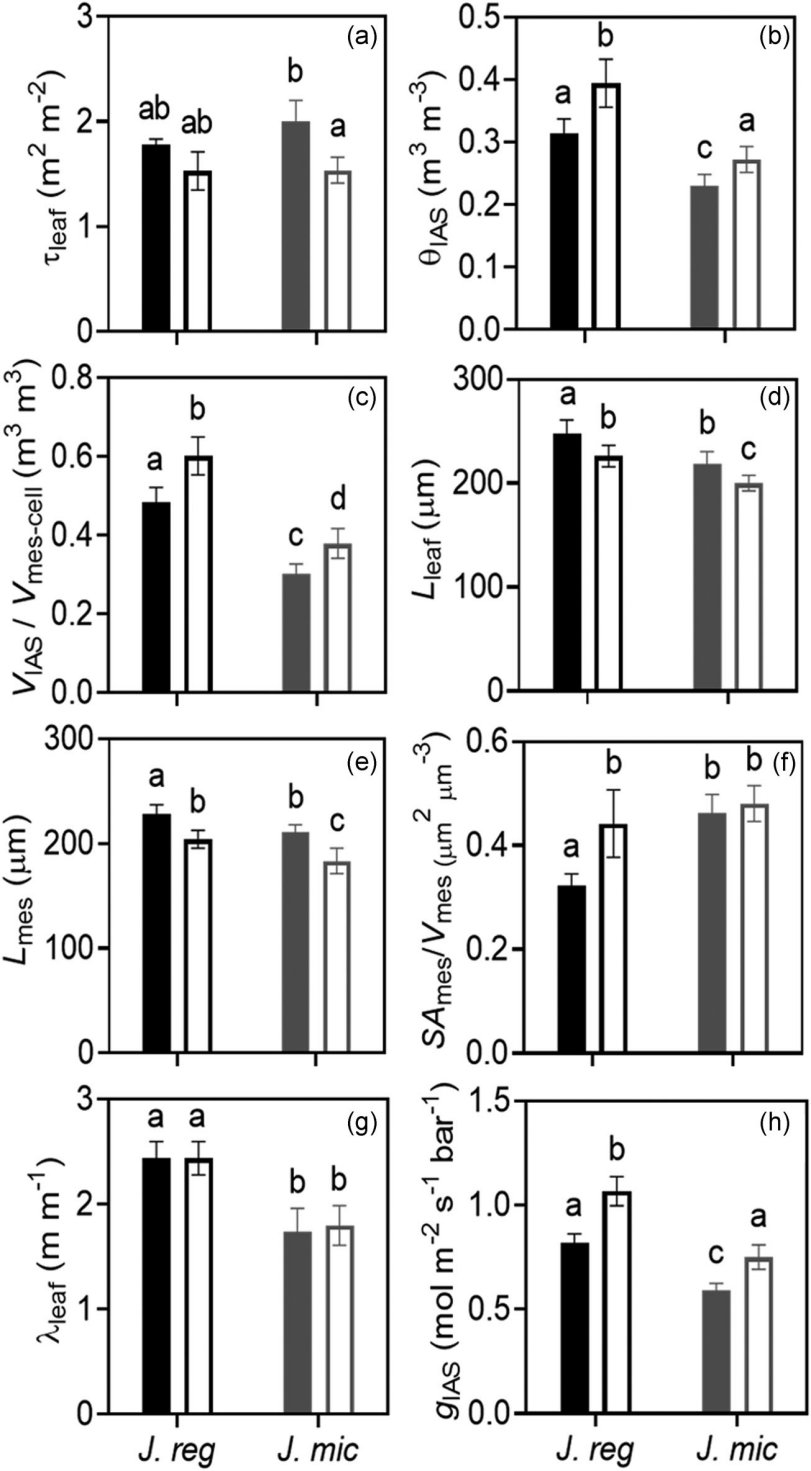
Intercellular airspace parameters under well‐watered (solid bar) and dehydrated (empty bar) treatments for *Juglans regia* and *Juglans microcarpa*. a, leaf tortuosity factor (τ_leaf_, m^2^ m^−2^); b, intercellular airspace porosity (θ_IAS_, m^3^ m^−^^3^); c, intercellular airspace to mesophyll cell volume ratio (*V*_IAS_/*V*_mes‐cell_); d, leaf thickness (*L*_leaf_, μm); e, mesophyll thickness (*L*_mes_, μm); f, mesophyll surface area exposed to the intercellular airspace per mesophyll volume basis (*SA*_mes_/*V*_mes_, μm^2^ μm^−^^3^); g, lateral path lengthening within intercellular airspace (*λ*_leaf_, m m^−1^); h, intercellular airspace conductance (*g*_IAS_, mol m^−2^ s^−1^ bar^−^^1^). Data points are means of four biological replicates (ramets) per species under well‐watered or dehydrated conditions (±SE)

Total leaf thickness (*L*_leaf_; *p* = 0.0183), mesophyll thickness (*L*_mes_; *p* = 0.0203), θ_IAS_ (*p* < 0.0001), *V*_IAS_/*V*_mes‐cell_, *g*_IAS_ (*p* < 0.0001) and *λ*_leaf_ (*p* = 0.0023) were greater in *J. regia* compared to *J. microcarpa* under well‐watered conditions (Figure 3). Lower θ_IAS_ in *J. microcarpa* aligned with significantly greater *SA*_mes_/*V*_mes_ (*p* < 0.0001, Figure 3). Tortuosity (*τ*_leaf_) (Figure 3) and adaxial and abaxial epidermis thicknesses (data not shown) were not statistically different between the species. Water stress reduced *L*_leaf_ (by 8% vs. 9%) and *L*_mes_ (by 10% vs. 13%) similarly in *J. regia* and *J. microcarpa*, respectively. Although the abaxial epidermis showed some shrinkage under dehydration, the abaxial and adaxial epidermis thickness (*L*_epi‐abaxial_, *L*_epi‐adaxial_) were not significantly reduced in either species. Dehydration increased *V*_IAS_/*V*_mes‐cell_ in both species by 20% (*p* < 0.0001) through reducing both *V*_IAS_ and *V*_mes‐cell_ but in different rates in each species (*p* < 0.0001, Figure 3). The reductions were in line with an increase in porosity (θ_IAS_) in both species under dehydration, but this effect was greater for *J. regia* than *J. microcarpa* (*p* = 0.0065) and significantly higher *SA*_mes_/*V*_mes_ under drought, in *J. regia* only (*p* = 0.010) (Figure 3). *g*_IAS_ increased equally for *J. regia* (by 23%) and *J. microcarpa* (by 21%) (*p* > 0.05) under dehydration compared to the well‐watered condition. Dehydration reduced *g*_IAS_ contribution to *g*_m_ (calculated as described by Niinemets & Reichstein, 2003) from 22% to 9% in *J. regia*, and 23% to 8% in *J. microcarpa* (*p* < 0.05). Although there was a significant increase in *τ*_leaf_ (by 23%) in *J. microcarpa* under dehydration (*p* = 0.010) (Figure 3), path lengthening (*λ*_leaf_) did not change in either species.

### 3.2 CO_2_ and light response curves

**Figure 4:**
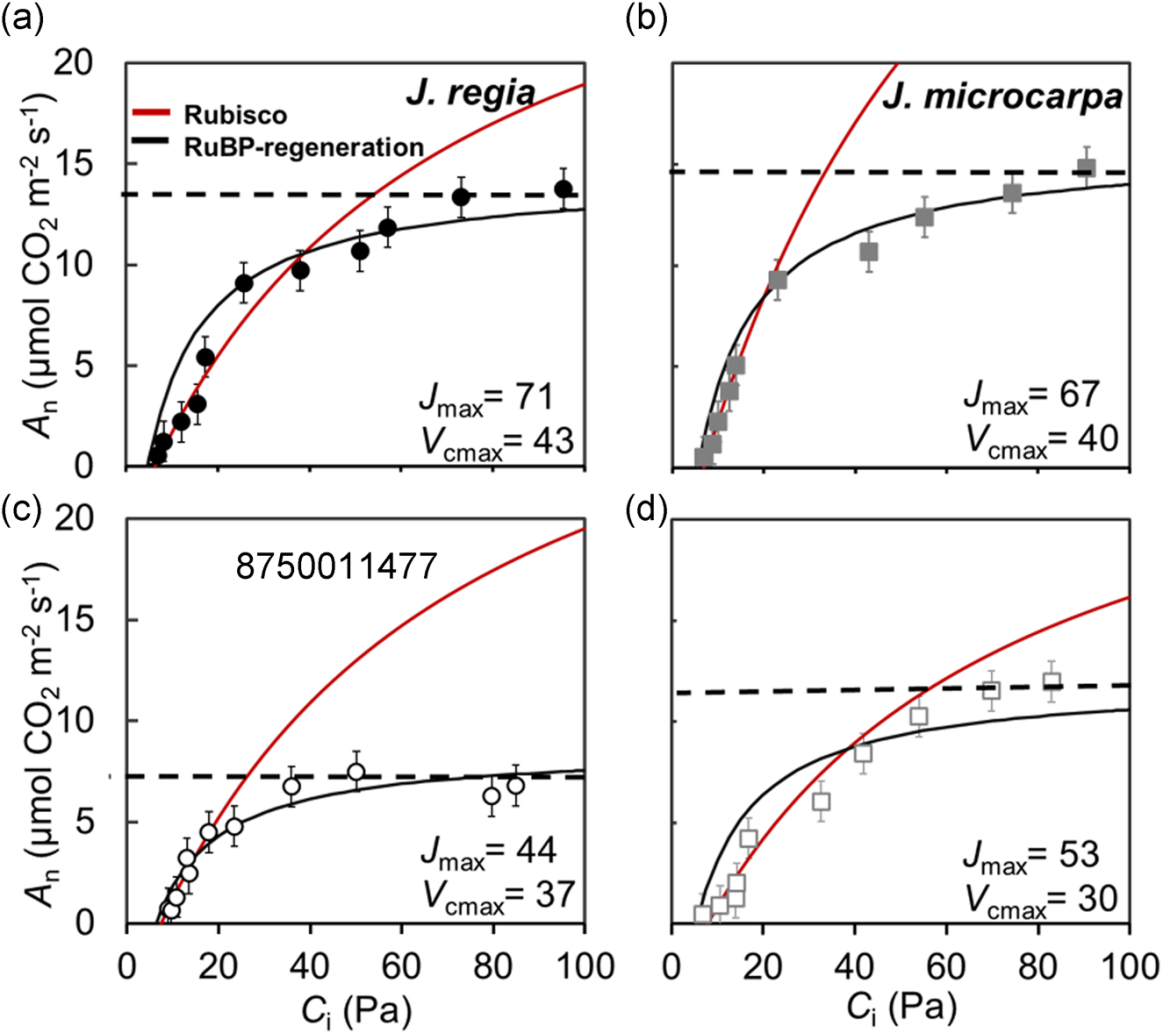
Photosynthetic CO_2_ response curves, relationship between mean *A*_n_ (net assimilation rate) and *C*_i_ (intercellular airspace CO_2_ concentration) at 1500 µmol m^−2^ s^−1^ photosynthetic photon flux density were constructed using FvCB model (Sharkey, 2016), averaged over four replications in *Juglans regia* (panels a and c) and *Juglans microcarpa* (panels b and d) under well‐watered (solid symbols and top row) and dehydrated (empty symbols and bottom row) treatments (±SE; *n* = 4). Assimilation rate at saturating CO_2_ (*A*_max_), Rubisco and RuBP regeneration limitations are indicated for each species [Color figure can be viewed at wileyonlinelibrary.com]

**Figure 5:**
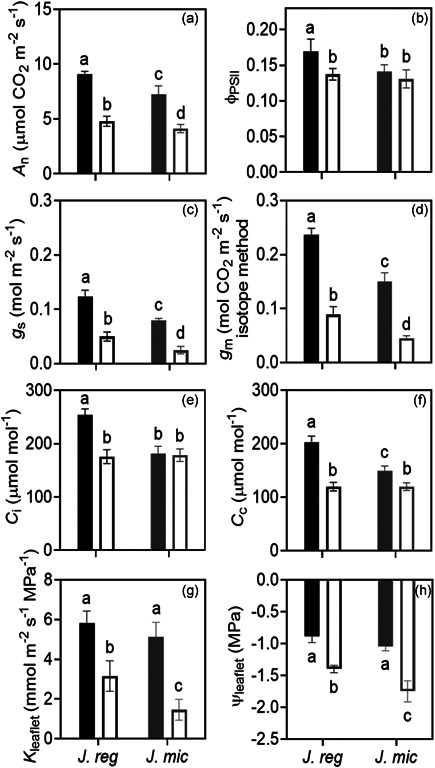
Photosynthetic traits under well‐watered (solid bar) and dehydrated (empty bar) treatments for *Juglans regia* (*J. reg*) and *Juglans microcarpa* (*J. mic*). a, net assimilation rate (*A*_n_, µmol CO_2_ m^−2^ s^−1^); b, photosystem II efficiency (Φ_PSII_); c, stomatal conductance (*g*_s_, mol m^−2^ s^−1^); d, mesophyll conductance (*g*_m_, mol CO_2_ m^−^^2^ s^−1^); e, intercellular airspace CO_2_ (C_i_, µmol mol^−1^); f, chloroplast CO_2_ (*C*_c_, µmol mol^−^^1^); g, leaflet hydraulic conductance (*K*_leaflet_, mmol m^−2^ s^−1^ MPa^−1^); h, leaflet water potential (Ψ_leaf_, MPa). Data points are means of four biological replicates (ramets) per species under well‐watered or dehydrated conditions (±SE). The measurements were taken at 400 µmol mol^−1^ and 1500 µmol m^−2^ s^−1^ photosynthetic photon flux density

Despite species‐dependent differences in photosynthetic capacity and greater *A*_n_ at ambient CO_2_ (400 µmol mol^−1^) and higher maximum carboxylation rate (*V*_cmax_) and maximum electron transport rate (*J*_max_) in *J. regia* as expected (Figures 4 and 5), maximum photosynthesis (*A*_max_ at *C*_i_ greater than 750 µmol mol^−1^) was statistically similar for the two species under well‐watered conditions (Figure 4). *Juglans microcarpa* maintained its photosynthetic capacity (i.e., greater *J*_max_) under dehydration to a greater extent compared to *J. regia* (17% vs. 52% decrease in *A*_max_, respectively; *p* < 0.0001). Dehydration reduced *A*_n_ significantly in both species (*p* = 0.0003) with a greater percent decrease in *J. regia* (by 47%) compared to a 42% for *J. microcarpa* (*p* = 0.0023) under ambient CO_2_ and saturating light (1500 µmol m^−2^ s^−1^) (Figure 4). At lower PPFD (50 to 500 µmol m^−2^ s^−1^) from adaxial illumination, the percent and absolute reductions in *A*_n_ were similar between the species (Figure S2). In general, *A*_n_ was lower with abaxial illumination, however, absolute and percent reductions in *A*_n_ were similar to those from adaxial illumination in the two species (*p* = 0.0014; Figure S2).

### 3.3 Mesophyll conductance and photosynthesis at ambient CO_2_

*A*_n_ and photosystem efficiency (Φ_PSII_) at ambient CO_2_ were greater in *J. regia* than *J. microcarpa* under control conditions (Figures 5a and 4b, *p* < 0.0083), in agreement with higher *g*_s_ (*p* = 0.0080; Figure 5c), *g*_m_ (*p* < 0.0001, stable isotope method, Figure 5d), *C*_i_ (*p* = 0.0001; Figure 5e), and *C*_c_ (*p* < 0.0001, Figure 5f). Reduced *A*_n_ under dehydration aligned with reductions in *g*_s_ and *g*_m_ (Figure 5). On the other hand, Φ_PSII_ and *C*_i_ decreased significantly in *J. regia* only and *C*_c_ showed greater reductions with dehydration in *J. regia* compared to *J. microcarpa* (Figure 5, *p*  < 0.0001). The stomatal aperture ratio (inner pore width/inner pore length) was greater for *J. regia* under well‐watered condition (*J. regia* 0.47 ± 0.04, *J. microcarpa* 0.37 ± 0.03), and dehydration induced stomatal closure and increased the ratio by 38% in *J. regia* versus 61% in to *J. microcarpa*. The relative changes in the pore dimensions were proportional to *g*_s_ reduction in *J. microcarpa* (by 68%), but less than reduction in *J. regia* (by 58%).

**Figure 6:**
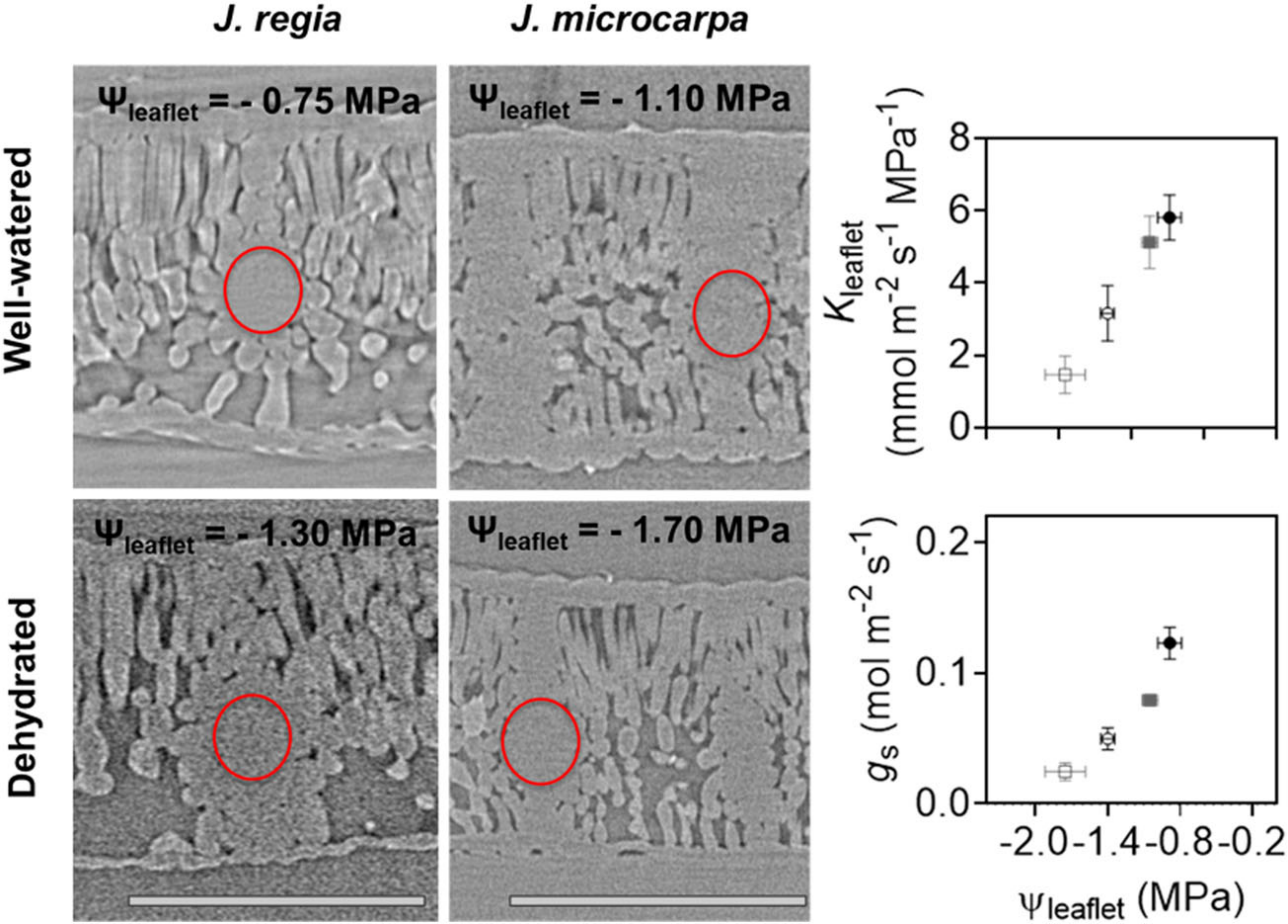
Leaflet water potential (Ψ_leaf_, MPa) and leaflet hydraulic conductance (*K*_leaflet_, mmol m^−2^ s^−1^ MPa^−1^) (a) and stomatal conductance (*g*_s_, mol m^−2^ s^−1^) (b) relationship under well‐watered (solid) and dehydrated (empty) treatments in *Juglans regia* (circle) and *Juglans microcarpa* (square). MicroCT images on the left are representative slices comparing conduit embolism from scans of *J. regia* and *J. microcarpa* leaves under well‐watered and dehydrated conditions. Six slices from each microCT scan were used to count number of emboli per secondary and tertiary veins under well‐watered and dehydrated conditions. Number of emboli divided by number of veins within the red circles region did not show a significant change in either secondary or tertiary veins under dehydration from well‐watered condition. Bar equals 250 μm [Color figure can be viewed at wileyonlinelibrary.com]

Under well‐watered conditions, Ψ_leaflet_ (Figure 5h) and *K*_leaflet_ (Figure 5g) were similar for the two species, however, they were correlated negatively in both species (*R* ^2^ = 0.9985, *p*  = 0.0008). *g*_s_ responded negatively to decreasing Ψ_leaflet_ (*R* ^2^ = 0.9091, *p* = 0.0465) with a greater reduction in *J. microcarpa* compared to *J. regia* (*p* < 0.0001) (Figure 6), however, the reductions in *K*_leaflet_ induced by water stress were not linked with significant changes in the number of embolized conduits for either species (Figure 6). The percent ratio of embolized conduits (*C*_emb_) per number of conduits (*C*) (Scoffoni et al., 2017) in representative microCT images (800 μm of each cross; *n* = 6) in secondary veins was similar between under well‐watered and dehydrated conditions in *J. regia* (11.3% vs. 12.4%) and *J. microcarpa* (20.2% vs. 14.2%) (*p* > 0.05). Similarly, no significant effect on emboli formation was found in tertiary veins under well‐watered versus dehydrated conditions in *J. regia* (7.4% vs. 10.9%) and *J. microcarpa*, (3.6% vs. 4.6%) (*p* > 0.05).

### 3.4 Chlorophyll distribution, light absorption and porosity profiles

**Figure 7:**
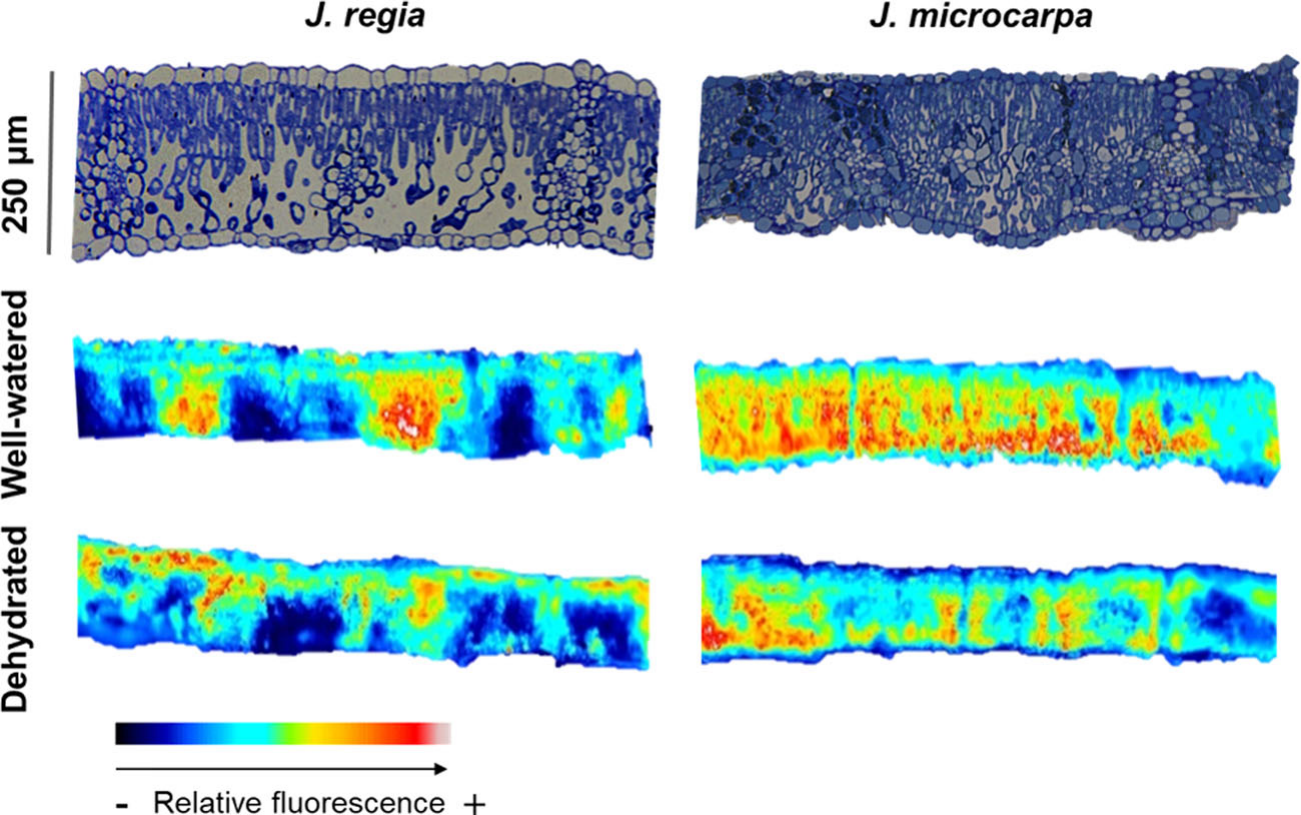
Top images compare microscopic cross sections for *Juglans regia* and *Juglans microcarpa* under well‐watered conditions. Bottom images compare spatial chlorophyll distribution from original epi‐illumination imaging and false‐coloured associated pairs, representative of the two species leaves under well‐watered and dehydrated conditions [Color figure can be viewed at wileyonlinelibrary.com]

**Figure 8:**
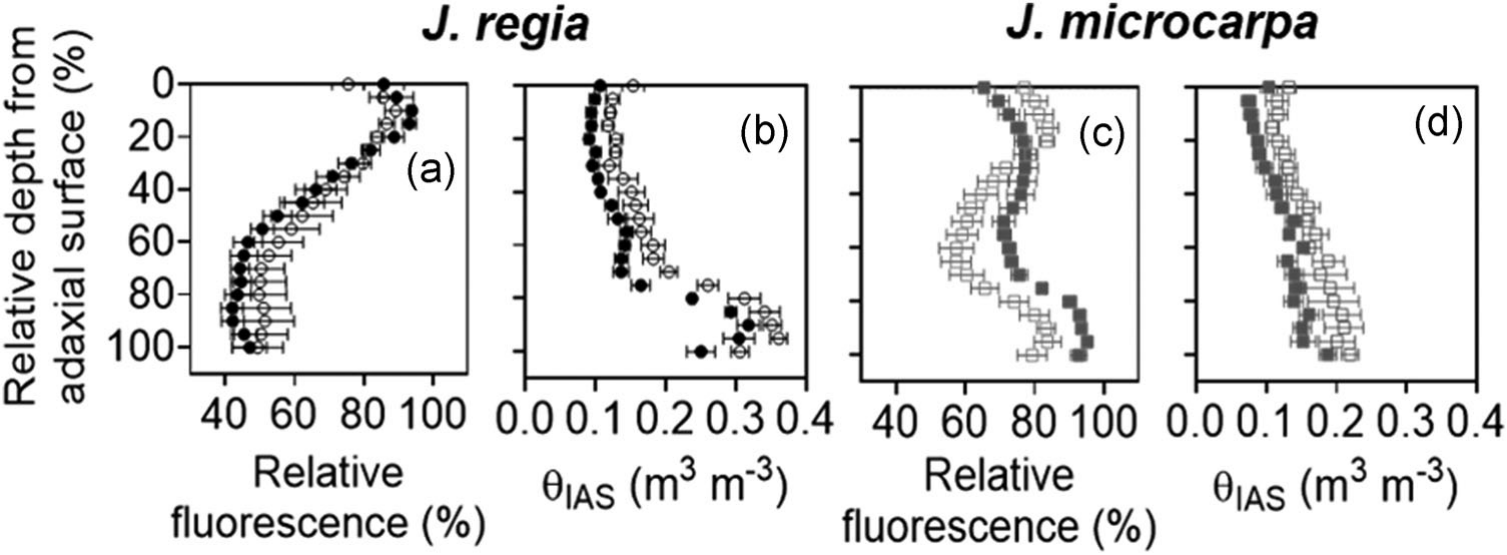
Chlorophyll fluorescence profiles obtained from epi‐illumination (490 nm) and porosity profiles acquired from microCT scans in *Juglans regia* (circles) and *Juglans microcarpa* (squares) under well‐watered (solid) and dehydrated (empty) treatments (mean ± SE; *n* = 4–6)

Relative chlorophyll distribution was estimated from fluorescence profiles in leaf cross sections using epi‐illumination (Figures 7 and S5). The patterns showed species‐specific differences; *J. regia* exhibited a single peak in relative fluorescence around palisade mesophyll, within 0%–20% depth from the adaxial epidermis (Figure 8a), whereas *J. microcarpa* had double peaks at 10%–40% and 80%–100% depth (Figure 8c). A rapid attenuation after 20% and leveling off at 60% of the depth in *J. regia* was different than the pattern for *J. microcarpa*, where there was a depression between 40% and 80% of leaf depth. As expected, porosity increased with depth from adaxial surface in two species, but the porosity profile complemented the fluorescence profile better in *J. regia* with an increase in porosity after 40% of depth, around spongy mesophyll, and a maximum between 90% adaxial depth (Figure 8b). In *J. microcarpa*, porosity changed less compared to relative fluorescence suggesting that components other than cell packing are involved in fluorescence gradients across the leaf. However, the porosity increased smoothly after 20% depth and reached the maximum between 80% and 100% depth from adaxial surface (Figure 8d).

**Figure 9:**
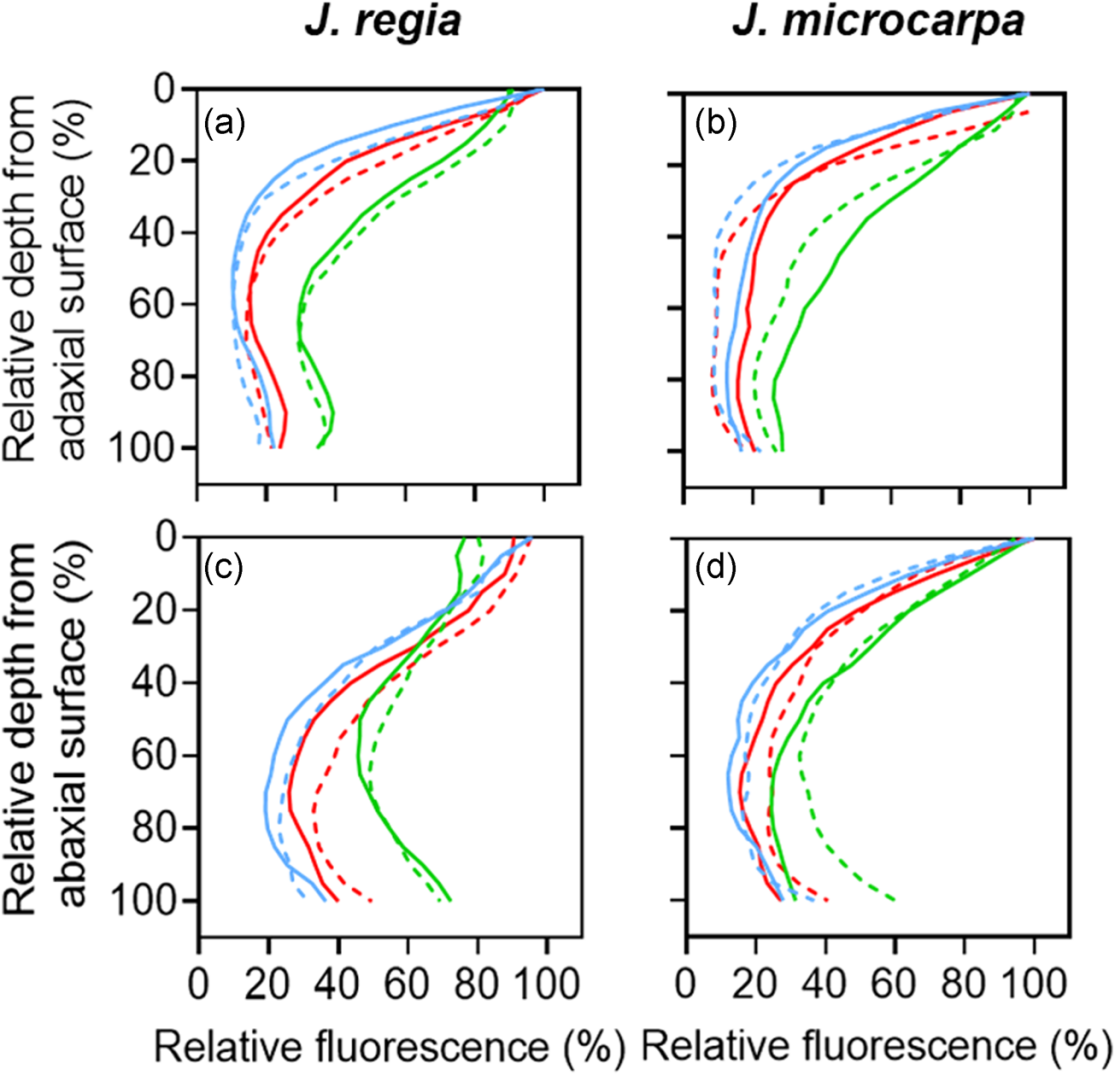
Light absorption profiles in *Juglans regia* and *Juglans microcarpa* leaves irradiated with direct monochromatic light at blue (488 nm), green (532 nm) and red (650 nm) wavelength under well‐watered (solid lines) and dehydrated (dashed lines) treatments averaged over four replications (*n* = 4). Directional lights were illuminated from adaxial (a and b) and abaxial (c and d) surfaces. Relative fluorescence (%) is presented relative to the illumination direction, adaxial (a and b) or abaxial (c and d) [Color figure can be viewed at wileyonlinelibrary.com]

Under dehydration, *J. microcarpa* showed an increase in relative fluorescence within the first 20% of the leaf depth and a consistent reduction between 30% and 100% depths (Figure 8c). In contrast, *J. regia* did not show a significant difference between watering conditions. Porosity increased across the leaf profile under dehydration in both species (*p* < 0.002) with a greater increase after 60% of adaxial depth in both species (Figure 8). Between 0% and 20% depth from the adaxial epidermis and under adaxial illumination, absorption of red light was greater in *J. microcarpa* under dehydration, but it decreased significantly after 30% depth compared to the well‐watered condition in all wavelengths (Figure 9). Only within 20% depth from the adaxial surface, *J. regia* showed higher absorption under dehydration at the blue wavelength (Figure 9). Illumination direction had a significant impact on absorption depth in red and blue wavelengths; maximum absorption at adaxial irradiance occurred at first 30% of depth from adaxial surface, whereas it was at first 60% of depth from abaxial epidermis under abaxial illumination. There was no consistent difference in light absorption profiles between well‐watered and dehydrated conditions under abaxial illumination in *J. regia*, while the green wavelength showed a significantly higher absorption between 60% and 100% of depth in *J. microcarpa*.

### 3.5 Paradermal cells and bundle sheath extensions

**Figure 10:**
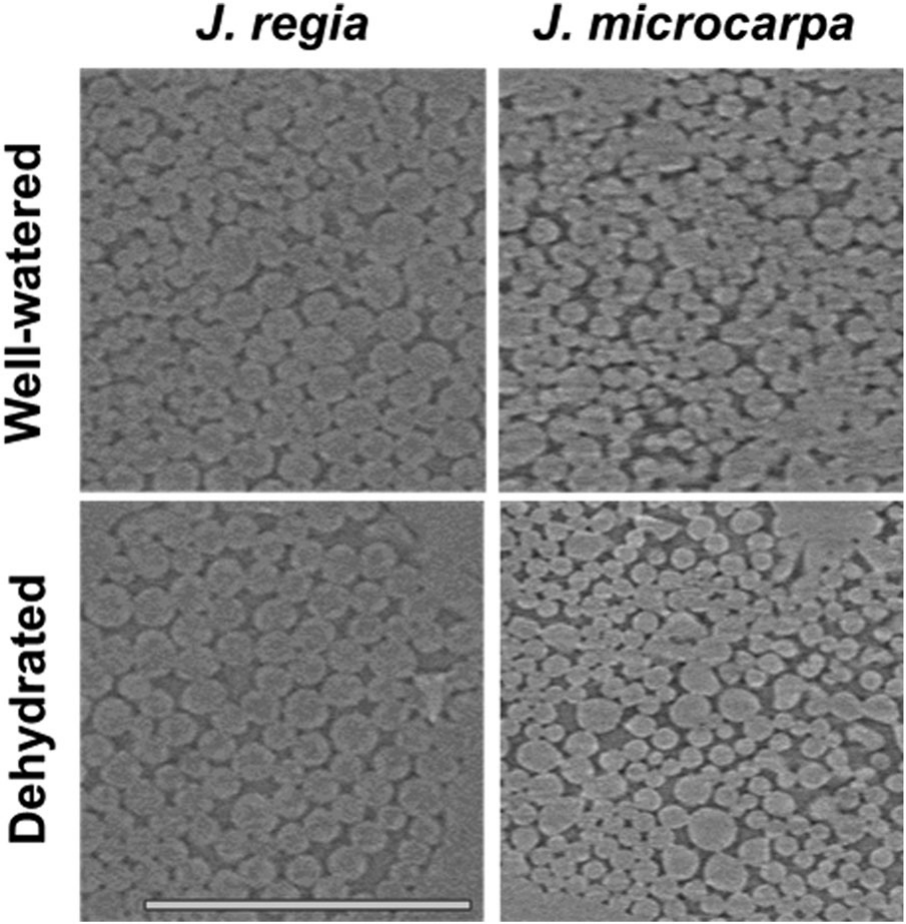
Paradermal sections at 20% depth from the adaxial surface in *Juglans regia* and *Juglans microcarpa* under well‐watered and dehydrated treatments. Similar responses were seen at 40% and 60% depths (images not shown). Bar equals 50 μm

**Figure 11:**
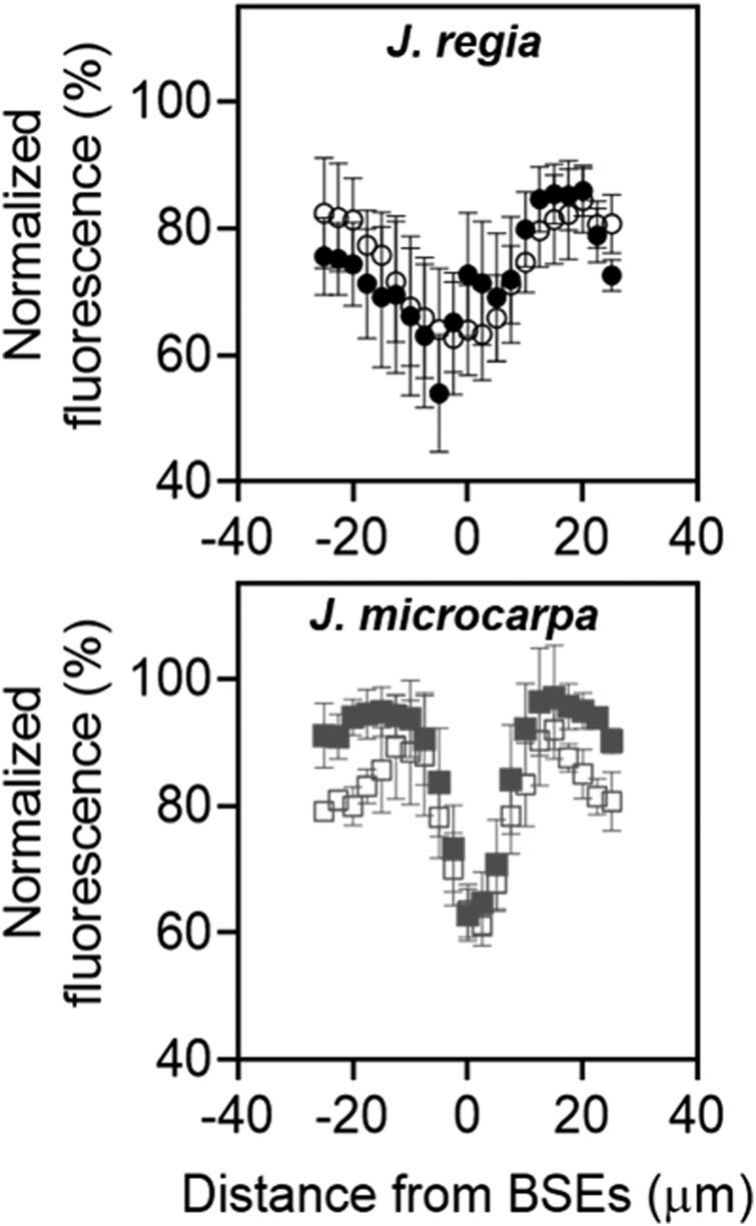
Fluorescence percentage normalised using maximum values per sample using epi‐illumination. It indicates the spatial distribution of fluorescence in equal horizontal distance from bundle sheath extensions (BSEs) in *Juglans regia* (circles—left data panel) and *Juglans microcarpa* (squares—right data panel) under well‐watered (solid) and dehydrated (empty) treatments (±SE; *n* = 4). Positive and negative values present variation in grayscale values at right or left side of BSEs. BSEs location is designated with white arrows; mean *J. regia* BSE width was ~4.7–5.0 μm and mean *J. microcarpa* BSE width was ~3.0–3.5 μm. Zero on the *x*‐axis of the data plots represents the centre of the measured BSE

The paradermal section images at 20%, 40% and 60% depths from the adaxial epidermal surface showed a significant decrease in the palisade cell diameter (μm) under dehydration in both species; diameters decreased by 14%, 9%, 15% in *J. microcarpa* and by 14%, 17%, 19% in *J. regia* (*p* < 0.05; see example images in Figure 10) at each increasing depth, respectively. BSEs were more prominent in *J. microcarpa* occupying 15% of the mesophyll volume compared to 8% in *J. regia* (*p* = 0.001). That was related to higher vein density with narrow BSEs width (Figure 11) for *J. microcarpa*. Using epi‐illumination data, *J. microcarpa* had higher fluorescence near the BSEs cells under well‐watered than dehydrated condition, compared to *J. regia*. (Figure 11, *p* < 0.0001). Under adaxial green wavelength illumination, differences in fluorescence near BSEs between the two species were not significant (normalised data shown in Figure S6).

## 4 DISCUSSION

### 4.1 Inherent differences between two *Juglans* species linked with structure and function

The exchange of water and CO_2_ and light absorption gradients are connected via mesophyll geometry and stress‐related changes in leaf anatomical characteristics induce responses in *g*_s_, *g*_m_, and *A*_n_. Using microCT imaging, we showed that θ_IAS_ and *g*_IAS_ in *J. regia* and *J. microcarpa* corresponded with species‐specific differences in *A*_n_, *g*_s_ and *g*_m_ obtained from gas exchange, chlorophyll fluorescence, and stable carbon isotope methods (Figures 3, 5 and S1). Well‐watered *J. regia*, with thicker leaves and denser mesophyll cells in the upper palisade, had higher θ_IAS_, *V*_IAS_/*V*_mes‐cell_ and *g*_IAS_ aligned with greater *A*_n_, *g*_s_ and *g*_m_ and higher chlorophyll concentration near the adaxial surface. *Juglans regia* mesophyll structure with higher θ_IAS_ and greater IAS distribution between mesophyll cells increase the *g*_IAS_ thorough an effective lateral diffusion (Figure 3). *J. regia* with less BSEs, forms less physical barrier to gas diffusion, thus the resistance to diffusion from gas to liquid phase decreases due to a greater lateral conductivity in this mesophyll type (e.g. homobaric) (Evans & von Caemmerer, 1996; Pieruschka et al., 2005). This inner mesophyll structure couples with a greater need for *g*_s_ to keep up with the higher demand for *C*_i_ concentration and higher *g*_smax_ (Figure 5). According to lateral CO_2_ diffusion modelling by Pieruschka et al. (2007), larger interconnected airspace can improve CO_2_ diffusivity through IAS, and enhance *C*_i_ in coordination with *g*_s_ as seen in *J. regia* (Figures 3 and 5). *Juglans regia* has larger stomata (average size, 122 vs. 72 μm^2^) but fewer stomata (62 per mm^2^) than *J. microcarpa* (79 per mm^2^), and higher λ_leaf_ in *J. regia* is linked with its lower stomatal density, which would increase CO_2_ diffusion length and higher θ_IAS_ near spongy mesophyll in hypostomatous species (similar to patterns reported by Earles et al., 2018 and Harwood et al., 2021). Therefore, *J. regia* benefits from a higher CO_2_ diffusion capacity, and exhibits improved performance through increasing maximum carboxylation rate (*V*_cmax_) and *A*_n_ under lower CO_2_ concentrations (Figure 4). At ambient CO_2_, where RUBP‐ regeneration is limiting, *J. regia* mesophyll with lower diffusion resistance (e.g., more porous leaves) and higher enzymatic activity during CO_2_ fixation and carbohydrate formation (i.e., Calvin cycle) shows greater maximum electron transport rate (*J*_max_), *g*_m_, and *A*_n_ under well‐watered condition.

Smaller mesophyll cell size potentially improves CO_2_ accessibility to Rubisco by enhancing *SA*_mes_/*V*_mes_ and chloroplast surface area (Ren et al., 2019; Terashima et al., 2006; Tholen et al., 2012), and consequently increases conductance within the liquid phase (*g*_liq_), while greater θ_IAS_ is associated with higher *g*_IAS_ (Théroux‐Rancourt et al., 2021). *g*_IAS_ contributed 29% and 25% to *g*_m_ in *J. regia* and *J. microcarpa*, respectively, consistent with previous findings in tree species; *Populus tremula* (23%–25%; Tosens et al., 2012), *Quercus ilex* L. (23%; Niinemets & Reichstein, 2003), and four *Eucalyptus* species (8%–21%; Harwood et al., 2021) under well‐watered condition and within the estimated limitation range by *g*_IAS_ (3%–37%) for *A*_n_ in hypostomatous species (Parkhurst & Mott, 1990). Higher *SA*_mes_/*V*_mes_ in well‐watered *J. microcarpa* was associated with lower θ_IAS_ and *V*_IAS_/*V*_mes‐cell_, and less variations in airspace distribution and relative fluorescence across leaf profiles (Figures 3 and 8). *Juglans microcarpa* mesophyll geometry increases resistances for CO_2_ diffusion in the gas phase (lower *g*_IAS_) through disconnecting mesophyll tissues and dividing into compartments and to the liquid phase, through potentially higher cell wall thickness (Pieruschka et al., 2008; Ren et al., 2019; Tomás et al., 2013), in line with less diffusion and carboxylation capacity, exhibited as *g*_m_, *V*_cmax_ and *J*_max_ responses for *J. microcarpa* (Figures 4 and 5). However, more biomass allocations toward cell packing and extensive bundle sheaths extensions, in species like *J. microcarpa*, improves the structural tolerance under the environmental stresses (e.g., low water) (Hikosaka & Shigeno, 2009; Niinemets et al., 2007).

Mesophyll cell packing and porosity distribution also led to different optical properties for the two *Juglans* species. *Juglans regia* leaves maintained greater θ_IAS_ under all conditions and mesophyll cells were more densely packed in the upper palisade. This coincided with maximum relative fluorescence at 10% depth and a decrease in fluorescence with increasing depth where large airspaces occurred in the spongy mesophyll layer (Figure 8), confirming our hypothesis for light absorption in *J. regia*. Well‐watered *J. regia* mesophyll had higher *A*_n_ and electron transport rate under variable light conditions (Figure S2), in agreement with our expectations for higher light absorption for a mesophyll with prominent palisade layers and high IAS volume in lower mesophyll (Cui et al., 1991; Gotoh et al., 2018), also reported for intermediate shade‐tolerant species (Hanba et al., 2002). This mesophyll structure appears to be an adaptation to maximize light absorption under varying light conditions (Leegood, 2008; Terashima, 1992; Tholen et al., 2012); as described by Vogelmann et al. (1996), larger IAS acts as “hall of mirrors” and improves absorption by multiple reflections between airspace, mesophyll and epidermal cells. *J. microcarpa*, on the other hand, had a narrower range of airspace distribution across leaf profiles with a smaller range of relative fluorescence that was distributed more evenly throughout the mesophyll, in line with mesophyll architecture for species from a high light environment (Hanba et al., 2002). The discrepancy between porosity and relative fluorescence profiles in *J. microcarpa* (Figure 8) could be attributed to light scattering inside the leaf due to other cell types (e.g., more BSEs) (Vogelmann & Evans, 2002), exhibited as significant difference in relative fluorescence near BSEs in two species under epi‐illumination (Figure 11). Mesophyll partitioning due to BSE presence (i.e., heterobaric leaves) is predicted to increase light penetration and overall absorption in various directions, mostly through spongy cells (Vogelmann & Martin, 1993; Xiao et al., 2016). More BSE volume in *J. microcarpa* compared to *J. regia* (15% vs. 8%, respectively) and greater fluorescence from the cells near the BSEs (Figure 11) under both well‐watered and dehydrated conditions indicate that *J. microcarpa* can utilise light deeper into the leaf (absolute data not shown). Species with BSEs that contain transparent cells with few or no chloroplasts are proposed to acclimate more effectively to drought since the light transmitted through BSEs is elevated at red and blue wavelengths and it may modify available internal light for photosynthetic tissues (Karabourniotis et al., 2000). BSE‐containing species like *J. microcarpa* may rely more on the structural support by BSEs than turgor associated with water supply to sustain their leaf stiffness (Read & Stokes, 2006) as an ecological response to water shortage in their growth habitat even if it costs a reduction in number of photosynthesising cells and eventually the carbon fixation.

### 4.2 Drought‐induced changes in photosynthetic capacity related to structure and function changes

Dehydration had a negative impact on both species by reducing *A*_n_, *g*_s_ and *g*_m_. *Juglans regia* was shown to be more susceptible to stress with a decline in Φ_PSII_ and a greater imbalance in energy distribution between PSII and PSI by more reduced distribution to PSII, also suggesting photodamage‐related decreases in light use efficiency under dehydration (Figure S2). Larger IAS volume increases evaporation surface for mesophyll cells, resulting in irreversible mesophyll cell shrinkage and potential permanent damage to the photosystems (Buckley et al., 2017; Rockwell et al., 2014c; Sack & Frole, 2006), as reflected by lower *A*_max_ for *J. regia* under saturating CO_2_ (Figure 4). A further reduction in *C*_i_ at low *g*_s_, concurrent with a decline in Φ_PSII_ suggests an increase in photorespiration (Medrano et al., 2002) as seen in *J. regia*. According to a sequential baseline presented by Trueba et al. (2019), *g*_s_ could decrease by 50% before TLP, whereas passing the TLP under severe stress can lead to permanent damage to the chlorophyll fluorescence as occurred in *J. regia*. This species exhibited a limited range of resilience to drought, as the widely cultivated walnut species in commercial nut production; this is consistent with irrigation management practices aimed at avoiding water stress in this species. In contrast, *J. microcarpa* with a more conservative water use strategy and a higher intrinsic water use efficiency (WUE_i_, 90 μmol CO_2_ mol^−1^ H_2_O vs. 76 μmol CO_2_ mol^−1^ H_2_O for *J. regia*) under the well‐watered condition, functions at lower water potentials under drought. The inherently lower *g*_s_ under well‐watered conditions and a greater reduction in Ψ_leaflet_ under dehydration for *J. microcarpa* suggests that this species may maintain low *g*_s_ at lower Ψ_leaflet_ closer to its turgor loss point (TLP). Species with prominent heterobaric leaves are also expected to show more nonuniform stomatal closure in response to environment stress (Kamakura et al., 2011). *Juglans microcarpa* is reported to be less vulnerable than *J. regia* to the xylem cavitation; it shows 12% decrease in stem xylem hydraulic conductivity at lower Ψ_xylem_ (e.g., ~ −1.6 vs. −1.3 MPa in *J. regia*) (Jinagool et al., 2018). Tyree et al. (1993) also measured about 50% reduction in petiole hydraulic conductivity for *J. regia* at Ψ_xylem_ ~ −1.4 MPa. Although TLP was not measured in our study, a greater increase in porosity and decrease in palisade cell diameter at less negative water potential suggest that *J. regia* functioned closer to its potential TLP under dehydration. These are commensurate with previous studies reporting reductions in mesophyll cell thickness and changes in IAS thickness near TLP to be species‐specific (Sancho‐Knapik et al., 2011; Scoffoni et al., 2014). In leaves with a structure like *J. microcarpa*, where the epidermis is more hydraulically integrated due to the presence of BSEs, water can bypass parts of the mesophyll reaching evaporation sites near epidermis as proposed by Zwieniecki et al. (2007). In this system, stomatal function is more closely linked to changes in xylem hydraulic conductance and stomata may show delays in the closure. Therefore, the relative presence of BSEs might play a role in allowing species to operate at more negative Ψ_xylem_.

Dehydration reduced *g*_s_ equally in both species, but decreased *K*_leaflet_ more severely in *J. microcarpa*, in relative terms (Figure 5). It has been shown that mutants lacking BSEs (e.g., *Solanum lycopersicum*) have lower *g*_s_, *A*_n_ and *K*_leaf_ than wild‐type plants (Zsögön et al., 2015). In addition, BSEs are proposed to slow down stomatal closure under stress‐induced conditions by enhancing hydraulic conductance through extravascular pathway (Barbosa et al., 2019; Buckley et al., 2011). Despite the higher presence of BSEs, the lower minimum *g*_s_ (0.02 mol H_2_O m^−2^ s^−1^) in *J. microcarpa* compared to *J. regia* (0.05 mol H_2_O m^−2^ s^−1^) may represent greater response through stomatal closure via highly reduced *K*_leaflet_ (driven by changes outside the xylem) at the expense of significantly lower *A*_n_ (Figures 5 and 6) and higher WUE_i_ at ambient CO_2_ under drought. Further, changes in *g*_s_ associated with stomatal aperture were supported by epidermal imprints, where stomatal aperture ratio decreased more for *J. microcarpa* (by 61%) compared to *J. regia* (by 38%). Dehydration reduced *g*_s_ to 27% and 11% of the *g*_smax_ for *J. regia* and *J. microcarpa*, respectively, further supporting the inherent difference in sensitivity of *g*_s_ to dehydration. Still, finding no significant emboli formation within xylem veins in either species (Figure 6) was in agreement with recent studies suggesting that declines in leaf hydraulic conductance are mostly due to declines in outside‐xylem tissue hydraulic conductance under mild to moderate dehydration and even beyond the TLP (Albuquerque et al., 2020; Scoffoni et al., 2017). Furthermore, the contradicting results for *J. microcarpa* may highlight the importance of aquaporins activity in changing *K*_leaf_ under induced conditions, such as a positive link between aquaporins abundance and *K*_leaf_ under high (vs. low) light was reported in *J. regia* (Cochard et al., 2007).

Drought‐induced shrinkage in mesophyll cells opens up more IAS volume within mesophyll, however nonuniform changes in cell shape can increase resistance to CO_2_ diffusion by reducing chloroplast surface area facing cell walls (Cano et al., 2013; Tosens et al., 2012; Xiao & Zhu, 2017). Dehydration increased porosity, leading to an increase in *g*_IAS_, however, the *g*_IAS_ contribution to *g*_m_ was reduced to 6‐8% in both species under dehydration (*p* < 0.05). Therefore, limitation imposed by *g*_liq_ to *g*_m_ may increase under drought via chloroplast re‐positioning and activity of CAs and aquaporins (Evans et al., 2009; Miyazawa et al., 2008; Momayyezi et al., 2020; Tholen et al., 2008; Tomás et al., 2013) more so than changes in resistance via anatomical components such as the cell wall thickness and cell wall composition (e.g., lignin deposition) (Evans, 2021; Roig‐Oliver et al., 2020). We must note, however, that the porous media approach of Earles et al. (2018) to compute *g*_IAS_, a step forward in the representation of the inherent 3D nature of the leaf mesophyll, does not fully account for the specificities of the diffusion within the leaf. As discussed by Harwood et al. (2021), path lengthening is a step forward to account for the discrete nature of stomata along the epidermis, but path shortening within the mesophyll would also occurs because of the gradient of carbon assimilation within the leaf profile, and would theoretically increase *g*_IAS_. In the present case, as water stress decreases photosynthesis, the path shortening effect could be smaller and might cancel out the *g*_IAS_ increases caused by higher porosity and lower tortuosity. Thus, the present result must be seen as a potential increase in *g*_IAS_ caused by anatomical changes.

Dehydration‐induced impacts on θ_IAS_ and mesophyll cell positioning altered the chlorophyll distribution in *J. microcarpa* by changing the magnitude and location of fluorescence peaks (Figure 8), in alignment with our expectations for *J. microcarpa* to highly reflect stress‐induced changes in cell geometry through light absorption. Increases in IAS volume between mesophyll cells, as seen by significant reductions in palisade cells diameter in paradermal sections at 20%, 40% and 60% from the adaxial epidermis (by 9% to 15%) (Figure 10), combined with increased frequency of BSEs in *J. microcarpa* to facilitate light diffusion and increase light absorption through the leaf and more at the spongy mesophyll under drought (Figure 9b). While dehydration reduced palisade cell diameter in *J. regia* at 40% and 60% adaxial depth, there was no significant change in absorption profiles (Figure 8) compared to the well‐watered leaves. This can further highlight the role of the spongy mesophyll arrangement (Borsuk et al., 2019) in light penetration and overall absorption efficiency through the leaf under stress.

## 5 CONCLUSIONS

Mesophyll structure has a substantial role in both CO_2_ diffusion and light absorption. *Juglans regia* mesophyll with thick palisade layers and higher IAS volume between mesophyll cells and mostly near the spongy layer, has higher *g*_m_ in line with more carboxylation capacity and greater light absorption under well‐watered condition. A more porous mesophyll with less BSEs has less anatomical leverage to tolerate dehydration and maintain the gas exchange in association with hydraulic components, and increases risk of damage to photosynthetic machinery. While more mesophyll cell density with less IAS distribution and greater BSEs (e.g., heterobaric leaf) can increase resistance to CO_2_ diffusion and lower overall light absorption and photosynthesis, it performs better in light absorption under drought. Greater BSEs in *J. microcarpa* leaves provide physical and hydraulic support leading to less mesophyll cell shrinkage with minimum damage to the carboxylation activity.

## CONFLICTS OF INTEREST

The authors declare no conflicts of interest.

## Supporting information

Supporting information.

## Data Availability

Data and materials are available on request from the corresponding author.

## References

[pce14287-bib-0001] Albuquerque, C. , Scoffoni, C. , Brodersen, C.R. , Buckley, T.N. , Sack, L. & McElrone, A.J. (2020) Coordinated decline of leaf hydraulic and stomatal conductances under drought is not linked to leaf xylem embolism for different grapevine cultivars. Journal of Experimental Botany, 71, 7286–7300. 33306796 10.1093/jxb/eraa392

[pce14287-bib-0002] Alonso‐Cantabrana, H. & von Caemmerer, S. (2015) Carbon isotope discrimination as a diagnostic tool for C_4_ photosynthesis in C_3_‐C_4_ intermediate species. Journal of Experimental Botany, 67, 3109–3121. 10.1093/jxb/erv555 PMC4867892 26862154

[pce14287-bib-0003] Barbosa, M.A.M. , Chitwood, D.H. , Azevedo, A.A. , Araújo, W.L. , Ribeiro, D.M. , Peres, L.E.P. et al. (2019) Bundle sheath extensions affect leaf structural and physiological plasticity in response to irradiance. Plant, Cell & Environment, 42, 1575–1589. 10.1111/pce.13495 30523629

[pce14287-bib-0004] Borsuk, A.M. & Brodersen, C.R. (2019) The spatial distribution of chlorophyll in leaves. Plant Physiology, 180, 1406–1417. 30944156 10.1104/pp.19.00094 PMC6752913

[pce14287-bib-0005] Borsuk, A.M. , Roddy, A.B. , Théroux‐Rancourt, G. & Brodersen, C.R. (2019) Emergent honeycomb topology of the leaf spongy mesophyll. bioRxiv 852459. Available from: 10.1101/852459

[pce14287-bib-0006] Brodersen, C.R. & Vogelmann, T.C. (2010) Do changes in light direction affect absorption profiles in leaves*?* Functional Plant Biology, 37, 403–412.

[pce14287-bib-0007] Brodribb, T.J. & Holbrook, N.M. (2003) Stomatal closure during leaf dehydration, correlation with other leaf physiological traits. Plant Physiology, 132, 2166–2173. 12913171 10.1104/pp.103.023879 PMC181300

[pce14287-bib-0008] Brodribb, T.J. , Powers, J. , Cochard, H. & Choat, B. (2020) Hanging by a thread? Forests and drought. Science, 368, 261–266. 32299945 10.1126/science.aat7631

[pce14287-bib-0009] Browne, G.T. , Leslie, C.A. , Grant, J.A. , Bhat, R.G. , Schmidt, L.S. , Hackett, W.P. et al. (2015) Resistance to species of *Phytophthora* identified among clones of *Juglans microcarpa* × *J. regia* . HortScience, 50, 1136–1142.

[pce14287-bib-0010] Buckley, T.N. (2019) How do stomata respond to water status? New Phytologist, 224, 21–36. 31069803 10.1111/nph.15899

[pce14287-bib-0011] Buckley, T.N. , John, G.P. , Scoffoni, C. & Sack, L. (2015) How does leaf anatomy influence water transport outside the xylem? Plant Physiology, 168, 1616–1635. 26084922 10.1104/pp.15.00731 PMC4528767

[pce14287-bib-0012] Buckley, T.N. , John, G.P. , Scoffoni, C. & Sack, L. (2017) The sites of evaporation within leaves. Plant Physiology, 173, 1763–1782. 28153921 10.1104/pp.16.01605 PMC5338672

[pce14287-bib-0013] Buckley, T.N. , Mott, K.A. & Farquhar, G.D. (2003) A hydromechanical and biochemical model of stomatal conductance. Plant, Cell & Environment, 26, 1767–1785.

[pce14287-bib-0014] Buckley, T.N. , Sack, L. & Gilbert, M.E. (2011) The role of bundle sheath extensions and life form in stomatal responses to leaf water status. Plant Physiology, 156, 962–973. 21459977 10.1104/pp.111.175638 PMC3177290

[pce14287-bib-0015] Canny, M. , Wong, S.C. , Huang, C. & Miller, C. (2012) Differential shrinkage of mesophyll cells in transpiring cotton leaves: implications for static and dynamic pools of water, and for water transport pathways. Functional Plant Biology, 39, 91–102. 32480764 10.1071/FP11172

[pce14287-bib-0016] Cano, F.J. , López, R. & Warren, C.R. (2014) Implications of the mesophyll conductance to CO_2_ for photosynthesis and water use efficiency during long‐term water stress and recovery in two contrasting Eucalyptus species. Plant, Cell & Environment, 37, 2470–2490. 10.1111/pce.12325 24635724

[pce14287-bib-0017] Cano, F.J. , Sánchez‐Gómez, D. , Rodríguez‐Calcerrada, J. , Warren, C.R. , Gil, L. & Aranda, I. (2013) Effects of drought on mesophyll conductance and photosynthetic limitations at different tree canopy layers. Plant, Cell & Environment, 36, 1961–1980. 10.1111/pce.12103 23527762

[pce14287-bib-0018] Carter, G.A. (1993) Responses of leaf spectral reflectance to plant stress. American Journal of Botany, 80, 239–243.

[pce14287-bib-0019] Carter, G.A. & Knapp, A.K. (2001) Leaf optical properties in higher plants: linking spectral characteristics to stress and chlorophyll concentration. American Journal of Botany, 88, 677–684. 11302854

[pce14287-bib-0020] Chaves, M.M. , Flexas, J. & Pinheiro, C. (2009) Photosynthesis under drought and salt stress: regulation mechanisms from whole plant to cell. Annals of Botany, 103, 551–560. 18662937 10.1093/aob/mcn125 PMC2707345

[pce14287-bib-0021] Choat, B. , Brodribb, T.J. , Brodersen, C.R. , Duursma, R.A. , López, R. & Medlyn, B.E. (2018) Triggers of tree mortality under drought. Nature, 558, 531–539. 29950621 10.1038/s41586-018-0240-x

[pce14287-bib-0022] Cochard, H. , Venisse, J.S. , Barigah, T.S. , Brunel, N. , Herbette, S. , Guilliot, A. et al. (2007) Putative role of aquaporins in variable hydraulic conductance of leaves in response to light. Plant Physiology, 143, 122–133. 17114274 10.1104/pp.106.090092 PMC1761984

[pce14287-bib-0023] Cowan, I.R. & Troughton, J.H. (1971) The relative role of stomata in transpiration and assimilation. Planta, 97, 325–336. 24493277 10.1007/BF00390212

[pce14287-bib-0024] Cui, M. , Vogelmann, T.C. & Smith, W.K. (1991) Chlorophyll and light gradients in sun and shade leaves of Spinacia oleracea. Plant, Cell & Environment, 14, 493–500.

[pce14287-bib-0025] Davis, T.J. , Gao, D. , Gureyev, T.E. , Stevenson, A.W. & Wilkins, S.W. (1995) Phase contrast imaging of weakly absorbing materials using hard X‐rays. Nature, 373, 595–598.

[pce14287-bib-0026] Dowd, B.A. , Campbell, G.H. , Marr, R.B. , Nagarkar, V.V. , Tipnis, S.V. & Axe, L. et al. (1999) Developments in synchrotron X‐ray computed microtomography at the National Synchrotron Light Source. In: Bonse, U. (Ed.) SPIE's International Symposium on Optical Science, Engineering, and Instrumentation. SPIE, pp. 224–236. 10.1117/12.363725

[pce14287-bib-0027] Earles, J.M. , Buckley, T.N. , Brodersen, C.R. , Busch, F.A. , Cano, F.J. , Choat, B. et al. (2019) Embracing 3D complexity in leaf carbon–water exchange. Trends in Plant Science, 24, 15–24. 30309727 10.1016/j.tplants.2018.09.005

[pce14287-bib-0028] Earles, J.M. , Théroux‐Rancourt, G. , Gilbert, M.E. , McElrone, A.J. & Brodersen, C.R. (2017) Excess diffuse light absorption in upper mesophyll limits CO_2_ drawdown and depresses photosynthesis. Plant Physiology, 174, 1082–1096. 28432257 10.1104/pp.17.00223 PMC5462040

[pce14287-bib-0029] Earles, J.M. , Théroux‐Rancourt, G. , Roddy, A.B. , Gilbert, M.E. , McElrone, A.J. & Brodersen, C.R. (2018) Beyond porosity: 3D leaf intercellular airspace traits that impact mesophyll conductance. Plant Physiology, 178, 148–162. 30042212 10.1104/pp.18.00550 PMC6130031

[pce14287-bib-0030] Evans, J.R. (1999) Leaf anatomy enables more equal access to light and CO_2_ between chloroplasts. New Phytologist, 143, 93–104.

[pce14287-bib-0031] Evans, J.R. (2021) Mesophyll conductance: walls, membranes and spatial complexity. New Phytologist, 229, 1864–1876. 33135193 10.1111/nph.16968

[pce14287-bib-0032] Evans, J.R. , Kaldenhoff, R. , Genty, B. & Terashima, I. (2009) Resistances along the CO_2_ diffusion pathway inside leaves. Journal of Experimental Botany, 60, 2235–2248. 19395390 10.1093/jxb/erp117

[pce14287-bib-0033] Evans, J.R. , Sharkey, T.D. , Berry, J.A. & Farquhar, G.D. (1986) Carbon isotope discrimination measured concurrently with gas exchange to investigate CO_2_ diffusion in leaves of higher plants. Australian Journal of Plant Physiology, 13, 281–292.

[pce14287-bib-0034] Evans, J.R. & Vogelmann, T.C. (2003) Profiles of ^14^C fixation through spinach leaves in relation to light absorption and photosynthetic capacity. Plant, Cell and Environment, 26, 547–560.

[pce14287-bib-0035] Evans, J.R. & von Caemmerer, S. (1996) Carbon dioxide diffusion inside Leaves. Plant Physiology, 110, 339–346. 12226185 10.1104/pp.110.2.339 PMC157726

[pce14287-bib-0036] Farquhar, G.D. & Cernusak, L.A. (2012) Ternary effects on the gas exchange of isotopologues of carbon dioxide. Plant, Cell and Environment, 35, 1221–1231. 10.1111/j.1365-3040.2012.02484.x 22292425

[pce14287-bib-0037] Farquhar, G.D. & Sharkey, T.D. (1982) Stomatal conductance and photosynthesis. Annual Review of Plant Physiology, 33, 317–345.

[pce14287-bib-0038] Franks, P.J. & Beerling, D.J. (2009) Maximum leaf conductance driven by CO_2_ effects on stomatal size and density over geologic time. Proceedings of the National Academy of Sciences United States of America, 106, 10343–10347. 10.1073/pnas.0904209106 PMC2693183 19506250

[pce14287-bib-0039] Flexas, J. , Barbour, M.M. , Brendel, O. , Cabrera, H.M. , Carriquí, M. , Díaz‐Espejo, A. et al. (2012) Mesophyll diffusion conductance to CO_2_: an unappreciated central player in photosynthesis. Plant Science, 193‐194, 70–84. 10.1016/j.plantsci.2012.05.009 22794920

[pce14287-bib-0040] Flexas, J. , Cano, F.J. , Carriquí, M. , Coopman, R. , Mizokami, Y. & Tholen, D. et al. (2018) CO_2_ diffusion inside photosynthetic organs. In: Adams, W.W., III & Terashima, I. (Eds.) Advances in photosynthesis and respiration: the leaf: a platform for performing photosynthesis and feeding the plant, 44. Springer International Publishing, pp. 163–208.

[pce14287-bib-0041] Flexas, J. , Ribas‐Carbó, M. , Diaz‐Espejo, A. , Galmés, J. & Medrano, H. (2008) Mesophyll conductance to CO_2_: current knowledge and future prospects. Plant, Cell and Environment, 31, 602–621. 10.1111/j.1365-3040.2007.01757.x 17996013

[pce14287-bib-0042] Galle, A. , Florez‐Sarasa, I. , Tomás, M. , Pou, A. , Medrane, H. , Ribas‐Carbo, M. et al. (2009) The role of mesophyll conductance during water stress and recovery in tobacco (*Nicotiana sylvestris*): acclimation or limitation? Journal of Experimental Botany, 60, 2379–2390. 19321646 10.1093/jxb/erp071

[pce14287-bib-0043] Galmés, J. , Abadía, A. , Cifre, J. , Medrano, H. & Flexas, J. (2007) Photoprotection processes under water stress and recovery in Mediterranean plants with growth forms and leaf habits. Physiologia Plantarum, 130, 495–510.

[pce14287-bib-0044] Genty, B. , Briantais, J.M. & Baker, N.R. (1989) The relationship between the quantum yield of photosynthetic electron transport and quenching of chlorophyll fluorescence. Biochimica et Biophysica Acta, 990, 87–92.

[pce14287-bib-0045] Gitelson, A.A. , Gritz, Y. & Merzlyak, M.N. (2003) Relationships between leaf chlorophyll content and spectral reflectance and algorithms for non‐destructive chlorophyll assessment in higher plant leaves. Journal of Plant Physiology, 160, 271–282. 12749084 10.1078/0176-1617-00887

[pce14287-bib-0046] Gommes, C.J. , Bons, A.J. , Blacher, S. , Dunsmuir, J.H. & Tsou, A.H. (2009) Practical methods for measuring the tortuosity of porous materials from binary or gray tone tomographic reconstructions. AIChE J, 55, 2000–2012.

[pce14287-bib-0047] Gotoh, E. , Suetsugu, N. , Higa, T. , Matsushita, T. , Tsukaya, H. & Wada, M. (2018) Palisade cell shape affects the light‐induced chloroplast movements and leaf photosynthesis. Scientific Reports, 8, 1472. 29367686 10.1038/s41598-018-19896-9 PMC5784166

[pce14287-bib-0048] Griffiths, H. , Weller, G. , Toy, L.F.M. & Dennis, R.J. (2013) You're so vein: bundle sheath physiology, phylogeny and evolution in C_3_ and C_4_ plants. Plant, Cell and Environment, 36, 249–261. 10.1111/j.1365-3040.2012.02585.x 22827921

[pce14287-bib-0049] Gürsoy, D. , De Carlo, F. , Xiao, X. & Jacobsen, C. (2014) TomoPy: a framework for the analysis of synchrotron tomographic data. Journal of Synchrotron Radiation, 21, 1188–1193. 25178011 10.1107/S1600577514013939 PMC4181643

[pce14287-bib-0050] Guy, R.D. , Fogel, M.L. & Berry, J.A. (1993) Photosynthetic fractionation of the stable isotopes of oxygen and carbon. Plant Physiology, 101, 37–47. 12231663 10.1104/pp.101.1.37 PMC158645

[pce14287-bib-0051] Hanba, Y.T. , Kogami, H. & Terashima, I. (2002) The effect of growth irradiance on leaf anatomy and photosynthesis in *Acer* species differing in light demand. Plant, Cell & Environment, 25, 1021–1030.

[pce14287-bib-0052] Harwood, R. , Théroux‐Rancourt, G. & Barbour, M.M. (2021) Understanding airspace in leaves: 3D anatomy and directional tortuosity. Plant, Cell & Environment, 44, 2455–2465. Available from: 10.1111/pce.14079 33974719

[pce14287-bib-0053] Hasey, J. (2016) Selecting the right clonal rootstock for managing soil and pest problems. University of California Agriculture and Natural Resources. Accessed: 2022. https://www.sacvalleyorchards.com/blog/walnuts-blog/selecting-the-right-clonal-rootstock-for-managing-soil-and-pest-problems/

[pce14287-bib-0054] Hikosaka, K. & Shigeno, A. (2009) The role of Rubisco and cell walls in the interspecific variation in photosynthetic capacity. Oecologia, 160, 443–51. 19288136 10.1007/s00442-009-1315-z

[pce14287-bib-0055] Holloway‐Phillips, M. (2019) Illuminating photosynthesis in the mesophyll of diverse leaves. Plant Physiology, 180, 1256–1258. 31253750 10.1104/pp.19.00592 PMC6752918

[pce14287-bib-0056] Hsiao, T.C. (1973) Plant responses to water stress. Annual Review of Plant Physiology, 24, 519–570.

[pce14287-bib-0057] Jinagool, W. , Lamacque, L. , Delmas, M. , Delzon, S. , Cochard, H. & Herbette, S. (2018) Is there variability for xylem vulnerability to cavitation in walnut tree cultivars and species (*Juglans* spp.)? HortScience, 53, 132–137.

[pce14287-bib-0058] Kamakura, M. , Kosugi, Y. , Takanashi, S. , Matsumoto, K. , Okumura, M. & Philip, E. (2011) Patchy stomatal behavior during midday depression of leaf CO_2_ exchange in tropical trees. Tree Physiology, 31, 160–168. 21383025 10.1093/treephys/tpq102

[pce14287-bib-0059] Karabourniotis, G. , Bornman, J.F. & Nikolopoulos, D. (2000) A possible optical role of the bundle sheath extensions of the heterobaric leaves of *Vitis vinifera* and *Quercus coccifera* . Plant, Cell and Environment, 23, 423–430.

[pce14287-bib-0060] Knipfer, T. , Reyes, C. , Momayyezi, M. , Brown, P.J. , Kluepfel, D. & McElrone, A.J. (2020) A comparative study on physiological responses to drought in walnut genotypes (RX1, Vlach, VX211) commercially available as rootstocks. Trees, 34, 665–678.

[pce14287-bib-0061] Koizumi, M. , Takahashi, K. , Mineuchi, K. , Nakamura, T. & Kano, H. (1998) Light gradients and the transverse distribution of chlorophyll fluorescence in mangrove and *Camellia* leaves. Annals of Botany, 81, 527–533.

[pce14287-bib-0062] Lanigan, G.J. , Betson, N. , Griffiths, H. & Seibt, U. (2008) Carbon isotope fractionation during photorespiration and carboxylation in *Senecio* . Plant Physiology, 148, 2013–2020. 18923019 10.1104/pp.108.130153 PMC2593675

[pce14287-bib-0063] Leegood, R.C. (2008) Roles of the bundle sheath cells in leaves of C_3_ plants. Journal of Experimental Botany, 59, 1663–1673. 18353763 10.1093/jxb/erm335

[pce14287-bib-0064] Legland, D. , Arganda‐Carreras, I. & Andrey, P. (2016) MorphoLibJ: integrated library and plugins for mathematical morphology with ImageJ. Bioinformatics, 32, 3532–3534. 27412086 10.1093/bioinformatics/btw413

[pce14287-bib-0065] McGranahan, G. & Leslie, C. (2009) Breeding walnuts (*Juglans regia*). In: Mohan Jain, S. & Priyadarshan, P.M. (Eds.) Breeding plantation tree crops: temperate species. Springer, pp. 249–273.

[pce14287-bib-0066] Medrano, H. , Escalona, J.M. , Bota, J. , Gulías, J. & Flexas, J. (2002) Regulation of photosynthesis of c_3_ plants in response to progressive drought: stomatal conductance as a reference parameter. Annals of Botany, 89, 895–905. 12102515 10.1093/aob/mcf079 PMC4233802

[pce14287-bib-0067] Miyazawa, S.I. , Yoshimura, S. , Shinzaki, Y. , Maeshima, M. & Miyake, C. (2008) Deactivation of aquaporins decreases internal conductance to CO_2_ diffusion in tobacco leaves grown under long‐term drought. Functional Plant Biology, 35, 553–564. 32688811 10.1071/FP08117

[pce14287-bib-0068] Momayyezi, M. & Guy, R.D. (2017a) Substantial role for carbonic anhydrase in latitudinal variation in mesophyll conductance of *Populus trichocarpa* Torr. & Gray. Plant, Cell and Environment, 40, 138–149. 10.1111/pce.12851 27761902

[pce14287-bib-0069] Momayyezi, M. & Guy, R.D. (2017b) Blue light differentially represses mesophyll conductance in high vs. low latitude genotypes of *Populus trichocarpa* Torr. & Gray. Journal of Plant Physiology, 213, 122–128. 28364640 10.1016/j.jplph.2017.03.006

[pce14287-bib-0070] Momayyezi, M. & Guy, R.D. (2018) Concomitant effects of mercuric chloride on mesophyll conductance and carbonic anhydrase activity in *Populus trichocarpa* Torr. & Gray. Trees, 32, 301–309.

[pce14287-bib-0071] Momayyezi, M. , McKown, A.D. , Bell, S.C.S. & Guy, R.D. (2020) Emerging roles for carbonic anhydrase in mesophyll conductance and photosynthesis. The Plant Journal, 101, 831–844. 31816145 10.1111/tpj.14638

[pce14287-bib-0072] Mott, K.A. & Peak, D. (2013) Testing a vapour‐phase model of stomatal responses to humidity. Plant, Cell & Environment, 36, 936–944. 10.1111/pce.12026 23072325

[pce14287-bib-0073] Muir, C.D. , Hangarter, R.P. , Moyle, L.C. & Davis, P.A. (2014) Morphological and anatomical determinants of mesophyll conductance in wild relatives of tomato (*Solanum* sect. *Lycopersicon*, sect. *Lycopersicoides*; Solanaceae). Plant, Cell and Environment, 37, 1415–1426. 10.1111/pce.12245 24279358

[pce14287-bib-0074] Nadal, M. & Flexas, J. (2018) Mesophyll conductance to CO_2_ diffusion: effects of drought and opportunities for improvement. In: García‐Tejero, I.F. & Durán‐Zuazo, V.H. (Eds.) Water scarcity and sustainable agriculture in semiarid environment, tools, strategies, and challenges for woody crops. Elsevier, Academic Press, pp. 403–438.

[pce14287-bib-0075] Niinemets, U. , Portsmuth, A. , Tena, D. , Tobias, M. , Matesanz, S. & Valladares, F. (2007) Do we underestimate the importance of leaf size in plant economics? Disproportional scaling of support costs within the spectrum of leaf physiognomy. Annals of Botany, 100, 283–303. 17586597 10.1093/aob/mcm107 PMC2735320

[pce14287-bib-0076] Niinemets, Ü. & Reichstein, M. (2003) Controls on the emission of plant volatiles through stomata: a sensitivity analysis. Journal of Geophysical Research, 108, 4211. Available from: 10.1029/2002JD002620

[pce14287-bib-0077] Oren, R. , Sperry, J.S. , Katul, G.G. , Pataki, D.E. , Ewers, B.E. , Phillips, N. et al. (1999) Survey and synthesis of intra‐ and interspecific variation in stomatal sensitivity to vapour pressure deficit. Plant, Cell & Environment, 22, 1515–1526.

[pce14287-bib-0078] Parkhurst, D.F. & Mott, K.A. (1990) Intercellular diffusion limits to CO_2_ uptake in leaves: studies in air and helox. Plant Physiology, 94, 1024–1032. 16667792 10.1104/pp.94.3.1024 PMC1077337

[pce14287-bib-0079] Pieruschka, R. , Schurr, U. & Jahnke, S. (2005) Lateral gas diffusion inside leaves. Journal of Experimental Botany, 56, 857–864. 15668225 10.1093/jxb/eri072

[pce14287-bib-0080] Pieruschka, R. , Chavarría‐Krauser, A. , Cloos, K. , Scharr, H. , Schurr, U. & Jahnke, S. (2007) Photosynthesis can be enhanced by lateral CO_2_ diffusion inside leaves over distances of several millimeters. New Phytologist, 178, 335–347. 10.1111/j.1469-8137.2008.02368.x 18312541

[pce14287-bib-0081] Pieruschka, R. , Chavarría‐Krauser, A. , Cloos, K. , Scharr, H. , Schurr, U. & Jahnke, S. (2008) Photosynthesis can be enhanced by lateral CO_2_ diffusion inside leaves over distances of several millimeters. New Phytologist, 178, 335–347. 18312541 10.1111/j.1469-8137.2008.02368.x

[pce14287-bib-0082] Pivovaroff, A. , Sharifi, R. , Scoffoni, C. , Sack, L. & Rundel, P. (2014) Making the best of the worst of times: traits underlying the combined shade and drought tolerance of *Ruscus aculeatus* and *Ruscus microglossum* (*Asparagaceae*). Functional Plant Biology, 41, 11–24. 10.1071/FP13047 32480962

[pce14287-bib-0083] Read, J. & Stokes, A. (2006) Plant biomechanics in an ecological context. American Journal of Botany, 93, 1546–1565. 21642101 10.3732/ajb.93.10.1546

[pce14287-bib-0084] Ren, T. , Weraduwage, S.M. & Sharkey, T.D. (2019) Prospects for enhancing leaf photosynthetic capacity by manipulating mesophyll cell morphology. Journal of Experimental Botany, 70, 1153–1165. 30590670 10.1093/jxb/ery448

[pce14287-bib-0085] Rockwell, F.E. , Michele & Holbrook, N. (2017) Leaf hydraulic architecture and stomatal conductance: a functional perspective. Plant Physiology, 174, 1996–2007. 28615346 10.1104/pp.17.00303 PMC5543976

[pce14287-bib-0086] Rockwell, F.E. , Michele Holbrook, N. & Stroock, A.D. (2014a) Leaf hydraulics I: Scaling transport properties from single cells to tissues. Journal of Theoretical Biology, 340, 251–266. 24112968 10.1016/j.jtbi.2013.09.036

[pce14287-bib-0087] Rockwell, F.E. , Michele Holbrook, N. & Stroock, A.D. (2014b) Leaf hydraulics II: Vascularized tissues. Journal of Theoretical Biology, 340, 267–284. 24012489 10.1016/j.jtbi.2013.08.027

[pce14287-bib-0088] Rockwell, F.E. , Michele Holbrook, N. & Stroock, A.D. (2014c) The competition between liquid and vapor transport in transpiring leaves. Plant Physiology, 164, 1741–1758. 24572172 10.1104/pp.114.236323 PMC3982738

[pce14287-bib-0089] Roig‐Oliver, M. , Bresta, P. , Nadal, M. , Liakopoulos, G. , Nikolopoulos, D. , Karabourniotis, G. et al. (2020) Cell wall composition and thickness affect mesophyll conductance to CO_2_ diffusion in *Helianthus annuus* under water deprivation. Journal of Experimental Botany, 71, 7198–7209. 32905592 10.1093/jxb/eraa413

[pce14287-bib-0090] Rueden, C.T. , Schindelin, J. , Hiner, M.C. , DeZonia, B.E. , Walter, A.E. & Arena, E.T. et al. (2017) ImageJ2: ImageJ for the next generation of scientific image data. BMC Bioinformatics, 18, 529. Available from: 10.1186/s12859-017-1934-z 29187165 PMC5708080

[pce14287-bib-0091] Rui, Y. & Anderson, C.T. (2016) Functional analysis of cellulose and xyloglucan in the walls of stomatal guard cells of Arabidopsis. Plant Physiology, 170, 1398–1419. 26729799 10.1104/pp.15.01066 PMC4775103

[pce14287-bib-0092] Sack, L. & Frole, K. (2006) Leaf structural diversity is related to hydraulic capacity in tropical rain forest trees. Ecology, 87, 483–491. 16637372 10.1890/05-0710

[pce14287-bib-0093] Sancho‐Knapik, D. , Alvarez‐Arenas, T.G. , Peguero‐Pina, J.J. , Fernández, V. & Gil‐Pelegrín, E. (2011) Relationship between ultrasonic properties and structural changes in the mesophyll during leaf dehydration. Journal of Experimental. Botany, 62, 3637–3645. 10.1093/jxb/err065 21414961

[pce14287-bib-0094] Scoffoni, C. , Albuquerque, C. , Brodersen, C.R. , Townes, S.V. , John, G.P. , Bartlett, M.K. et al. (2017) Outside xylem vulnerability, not xylem embolism, controls leaf hydraulic decline during dehydration. Plant Physiology, 173, 1197–1210. 28049739 10.1104/pp.16.01643 PMC5291720

[pce14287-bib-0095] Scoffoni, C. , Vuong, C. , Diep, S. , Cochard, H. & Sack, L. (2014) Leaf shrinkage with dehydration: coordination with hydraulic vulnerability and drought tolerance. Plant Physiology, 164, 1772–1788. 24306532 10.1104/pp.113.221424 PMC3982740

[pce14287-bib-0096] Sharkey, T.D. (2016) What gas exchange data can tell us about photosynthesis. Plant, Cell & Environment, 39, 1161–1163. 10.1111/pce.12641 26390237

[pce14287-bib-0097] Simonin, K.A. , Burns, E. , Choat, B. , Barbour, M.M. , Dawson, T.E. & Franks, P.J. (2015) Increasing leaf hydraulic conductance with transpiration rate minimizes the water potential drawdown from stem to leaf. Journal of Experimental Botany, 66, 1303–1315. 25547915 10.1093/jxb/eru481 PMC4339593

[pce14287-bib-0098] Smith, W.K. , Critchley, C. & Vogelmann, T.C. (2004) Photosynthetic adaptation from the chloroplast to the landscape. Ecological Studies. Springer‐Verlag.

[pce14287-bib-0099] Smith, W.K. , Vogelmann, T.C. , DeLucia, E.H. , Bell, D.T. & Shepherd, K.A. (1997) Leaf form and photosynthesis. BioScience, 47, 785–793.

[pce14287-bib-0100] Takahashi, K. , Mineuchi, K. , Nakamura, T. , Koizumi, M. & Kano, H. (1994) A system for imaging transverse distribution of scattered light and chlorophyll fluorescence in intact rice leaves. Plant, Cell & Environment, 17, 105–110.

[pce14287-bib-0101] Terashima, I. (1992) Anatomy of non‐uniform leaf photosynthesis. Photosynthesis Research, 31, 195–212. 24408060 10.1007/BF00035537

[pce14287-bib-0102] Terashima, I. , Hanba, Y.T. , Tazoe, Y. , Vyas, P. & Yano, S. (2006) Irradiance and phenotype: comparative eco‐development of sun and shade leaves in relation to photosynthetic CO_2_ diffusion. Journal of Experimental Botany, 57, 343–354. 16356943 10.1093/jxb/erj014

[pce14287-bib-0103] Théroux‐Rancourt, G. , Earles, J.M. , Gilbert, M.E. , Zwieniecki, M.J. , Boyce, C.K. , McElrone, A.J. et al. (2017) The bias of a two‐dimensional view: comparing two‐dimensional and three‐dimensional mesophyll surface area estimates using noninvasive imaging. New Phytologist, 215, 1609–1622. 28691233 10.1111/nph.14687

[pce14287-bib-0104] Théroux‐Rancourt, G. & Gilbert, M.E. (2017) The light response of mesophyll conductance is controlled by structure across leaf profiles. Plant, Cell and Environment, 40, 726–740. 10.1111/pce.12890 28039917

[pce14287-bib-0105] Theroux‐Rancourt, G. , Jenkins, M.R. , Brodersen, C.R. , McElrone, A. , Forrestel, E.J. & Earles, J.M. (2020) Digitally deconstructing leaves in 3D using X‐ray microcomputed tomography and machine learning. Applications in Plant Sciences, 8, e11380. 32765979 10.1002/aps3.11380 PMC7394714

[pce14287-bib-0106] Théroux‐Rancourt, G. , Roddy, A.B. , Earles, J.M. , Gilbert, M.E. , Zwieniecki, M.A. & Boyce, C.K. et al. (2021) Maximum CO_2_ diffusion inside leaves is limited by the scaling of cell size and genome size. Proceedings of the Royal Society B, 288, 20203145. Available from: 10.1098/rspb.2020.3145 33622134 PMC7934972

[pce14287-bib-0107] Tholen, D. , Boom, C. , Noguchi, K. , Ueda, S. , Katase, T. & Terashima, I. (2008) The chloroplast avoidance response decreases internal conductance to CO_2_ diffusion in *Arabidopsis thaliana* leaves. Plant, Cell & Environment, 31, 1688–1700. 10.1111/j.1365-3040.2008.01875.x 18721264

[pce14287-bib-0108] Tholen, D. , Boom, C. & Zhu, X.‐G. (2012) Opinion: prospects for improving photosynthesis by altering leaf anatomy. Plant Sciences, 197, 92–101. 10.1016/j.plantsci.2012.09.005 23116676

[pce14287-bib-0109] Tholen, D. & Zhu, X.G. (2011) The mechanistic basis of internal conductance: a theoretical analysis of mesophyll cell photosynthesis and CO_2_ diffusion. Plant Physiology, 156, 90–105. 21441385 10.1104/pp.111.172346 PMC3091052

[pce14287-bib-0110] Tomás, M. , Flexas, J. , Copolovici, L. , Galmés, J. , Hallik, L. , Medrano, H. et al. (2013) Importance of leaf anatomy in determining mesophyll diffusion conductance to CO_2_ across species: quantitative limitations and scaling up by models. Journal of Experimental Botany, 64, 2269–2281. 23564954 10.1093/jxb/ert086 PMC3654418

[pce14287-bib-0111] Tosens, T. & Laanisto, L. (2018) Mesophyll conductance and accurate photosynthetic carbon gain calculations. Journal of Experimental Botany, 69, 5315–5318. 30476280 10.1093/jxb/ery369

[pce14287-bib-0112] Tosens, T. , Niinemets, Ü. , Vislap, V. , Eichelmann, H. & Castro‐Díez, P. (2012) Developmental changes in mesophyll diffusion conductance and photosynthetic capacity under different light and water availabilities in *Populus tremula*: how structure constrains function. Plant, Cell & Environment, 35, 839–856. 10.1111/j.1365-3040.2011.02457.x 22070625

[pce14287-bib-0113] Tosens, T. , Nishida, K. , Gago, J. , Coopman, R.E. , Cabrera, H.M. , Carriqu, M. et al. (2016) The photosynthetic capacity in 35 ferns and fern allies: mesophyll CO_2_ diffusion as a key trait. New Phytologist, 209, 1576–1590. 26508678 10.1111/nph.13719

[pce14287-bib-0114] Trueba, S. , Pan, R. , Scoffoni, C. , John, G.P. , Davis, S.D. & Sack, L. (2019) Thresholds for leaf damage due to dehydration: declines of hydraulic function: stomatal conductance and cellular integrity precede those for photochemistry. New Phytologist, 223, 134–149. 30843202 10.1111/nph.15779

[pce14287-bib-0115] Turner, N.C. , Schulze, E.D. & Gollan, T. (1984) The responses of stomata and leaf gas exchange to vapour pressure deficits and soil water content I. Species comparisons at high soil water contents. Oecologia, 63, 338–342. 28311208 10.1007/BF00390662

[pce14287-bib-0116] Tyree, M.T. , Cochard, H. , Cruiziat, P. , Sinclair, B. & Ameglio, T. (1993) Drought‐induced leaf shedding in walnut: evidence for vulnerability segmentation. Plant, Cell & Environment, 16, 879–882.

[pce14287-bib-0118] Urban, L. , Aarrouf, J. & Bidel, L.P.R. (2017) Assessing the effects of water deficit on photosynthesis using parameters derived from measurements of leaf gas exchange and of chlorophyll a fluorescence. Frontiers in Plant Science, 8, 2068. Available from: 10.3389/fpls.2017.02068 29312367 PMC5735977

[pce14287-bib-0119] Vogelmann, T.C. & Evans, J.R. (2002) Profiles of light absorption and chlorophyll within spinach leaves from chlorophyll fluorescence. Plant, Cell and Environment, 25, 1313–1323.

[pce14287-bib-0120] Vogelmann, T.C. & Han, T. (2000) Measurement of gradients of absorbed light in spinach leaves from chlorophyll fluorescence profiles. Plant, Cell and Environment, 23, 1303–1311.

[pce14287-bib-0121] Vogelmann, T.C. & Martin, G. (1993) The functional significance of palisade tissue: penetration of directional versus diffuse light. Plant, Cell and Environment, 16, 65–72.

[pce14287-bib-0122] Vogelmann, T.C. , Nishio, J.N. & Smith, W.K. (1996) Leaves and light capture: light propagation and gradients of carbon fixation within leaves. Trends in Plant Science, 1, 65–70.

[pce14287-bib-0123] Williams, L.E. & Araujo, F. (2002) Correlations among predawn leaf, midday leaf, and midday stem water potential and their correlations with other measures of soil and plant water status in *Vitis vinifera* L. Journal of the American Society for Horticultural Sciences, 127, 448–454.

[pce14287-bib-0124] Wingate, L. , Seibt, U. , Moncrieff, J.B. , Jarvis, P.G. & Lloyd, J. (2007) Variations in ^13^C discrimination during CO_2_ exchange by *Picea sitchensis* branches in the field. Plant, Cell and Environment, 30, 600–616. 10.1111/j.1365-3040.2007.01647.x 17407538

[pce14287-bib-0125] Xiao, Y. , Tholen, D. & Zhu, X.G. (2016) The influence of leaf anatomy on the internal light environment and photosynthetic electron transport rate: exploration with a new leaf ray tracing model. Journal of Experimental Botany, 67, 6021–6035. 27702991 10.1093/jxb/erw359 PMC5100017

[pce14287-bib-0126] Xiao, Y. & Zhu, X.G. (2017) Components of mesophyll resistance and their environmental responses: a theoretical modelling analysis. Plant, Cell and Environment, 40, 2729–2742. 10.1111/pce.13040 28743156

[pce14287-bib-0127] Zhou, Y. , Lam, H.M. & Zhang, J. (2007) Inhibition of photosynthesis and energy dissipation induced by water and high light stresses in rice. Journal of Experimental Botany, 58, 1207–1217. 17283375 10.1093/jxb/erl291

[pce14287-bib-0128] Zsögön, A. , Alves Negrini, A.C. , Peres, L.E.P. , Nguyen, H.T. & Ball, M.C. (2015) A mutation that eliminates bundle sheath extensions reduces leaf hydraulic conductance, stomatal conductance and assimilation rates in tomato (*Solanum lycopersicum*). New Phytologist, 205, 618–626. 25267094 10.1111/nph.13084

[pce14287-bib-0129] Zwieniecki, M.A. , Brodribb, T.J. & Holbrook, N.M. (2007) Hydraulic design of leaves: insights from rehydration kinetics. Plant, Cell & Environment, 30, 910–921. 10.1111/j.1365-3040.2007.001681.x 17617819

