# Supplementary material for: Desiccation of the leaf mesophyll and its implications for CO2 diffusion and light processing
Source: Plant Cell Environ. 2022 Mar 3;45(5):1362–81. doi: 10.1111/pce.14287 (PMC9314819; doi:10.1111/pce.14287)
Supplement: Supplementary file 1 — Supporting information. [file PCE-45-1362-s001.docx]

**Supporting Information**

**Fig. S1** Correlation between mesophyll conductance (*g*_m_, mol CO_2_ m^-2^ s^-1^) obtained from stable carbon isotope discrimination and chlorophyll fluorescence methods (*n* = 16) of *J. regia* (circles) and *J. microcarpa* (squares) under well-watered (solid) and dehydrated (empty) treatments.


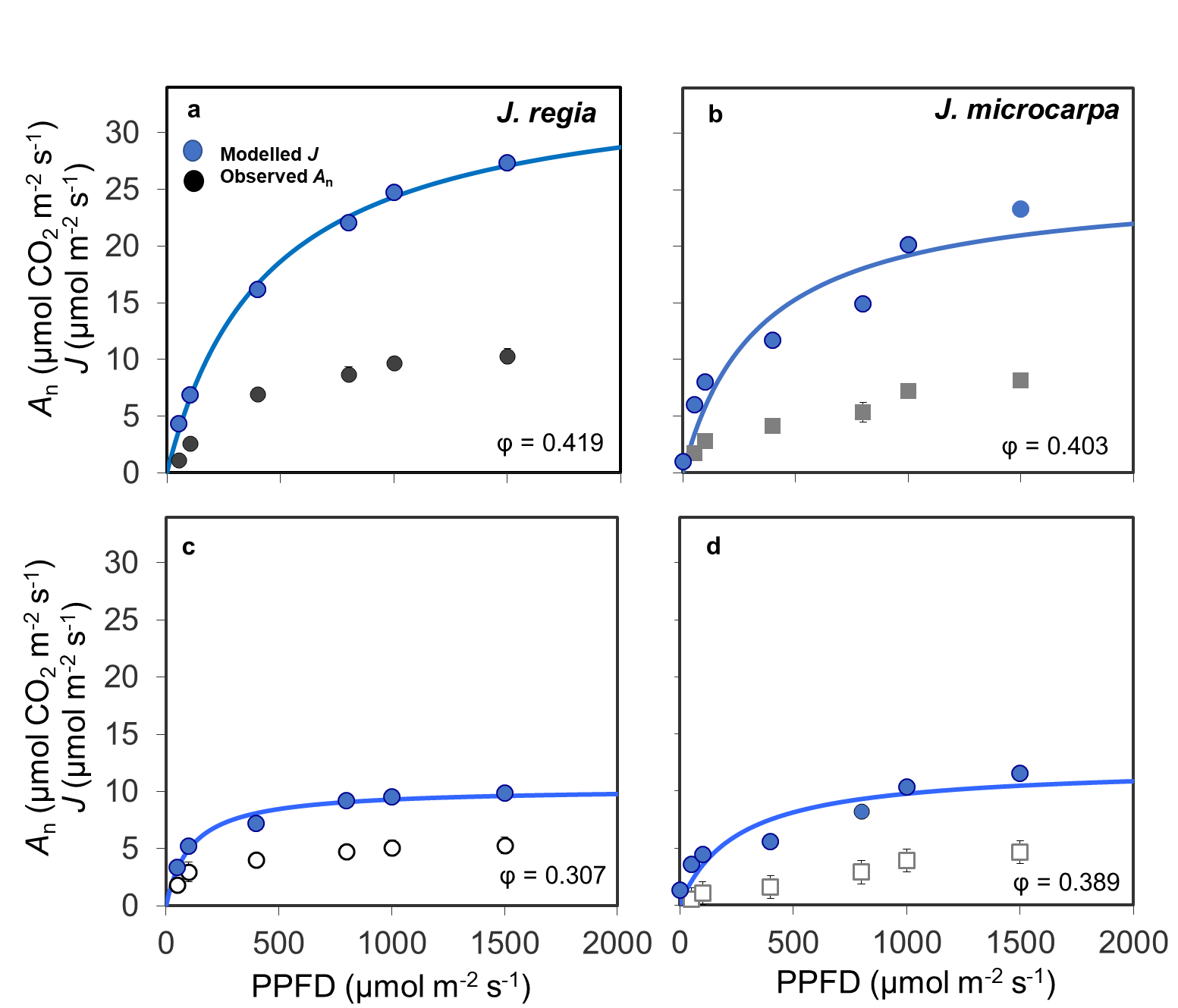


**Well-watered**

**Dehydrated**

**Fig. S2** Light response curves, relationship between *A*_n_ (net assimilation rate) and modelled *J* (electron transport rate) and PPFD at 400 µmol mol^-1^ (± SE; *n* = 4) from adaxial illumination, averaged over four replications in *J. regia* (panels a and c) and *J. microcarpa* (panels b and d) were constructed using FvCB model (Sharkey 2016) under well-watered (solid) and dehydrated (empty) treatments. φ is the initial slope of *A*_n_ versus light and represents fraction of the energy distributed between PSII vs. PSI.


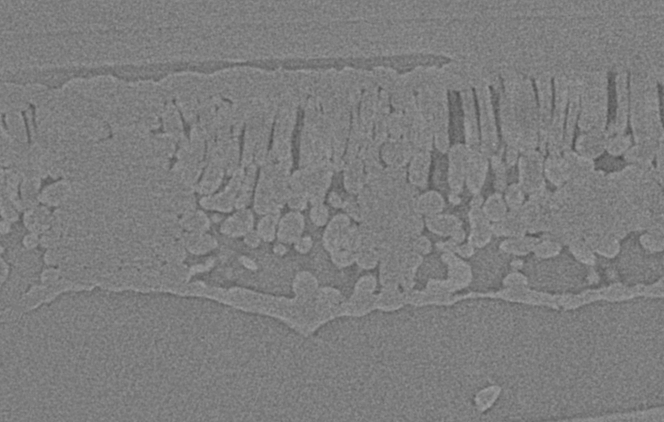

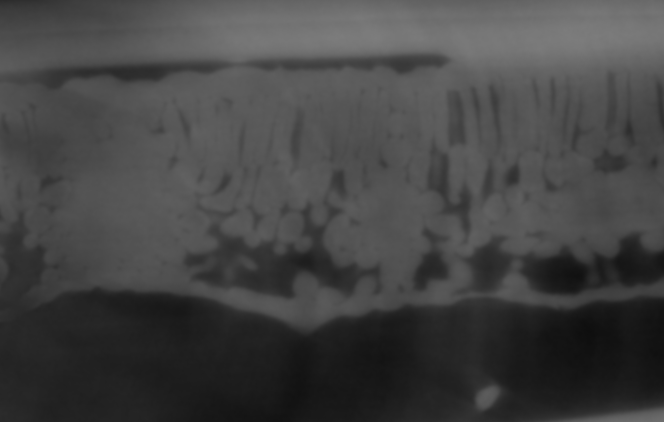

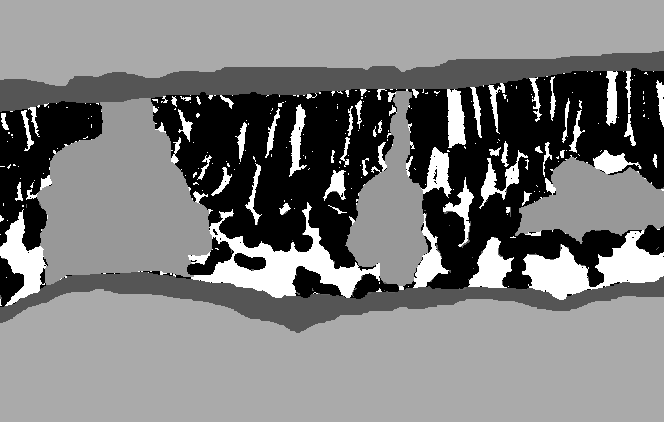

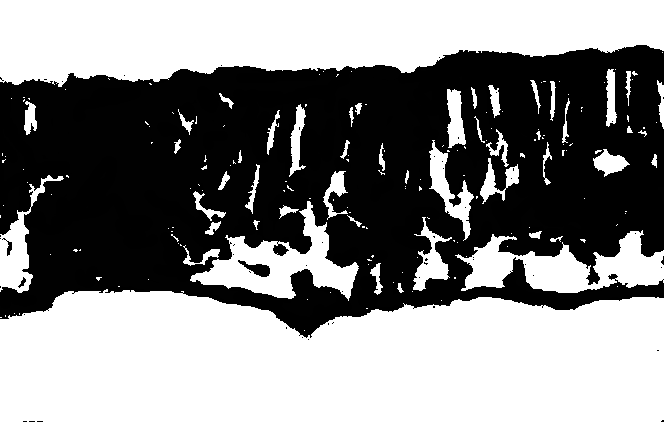

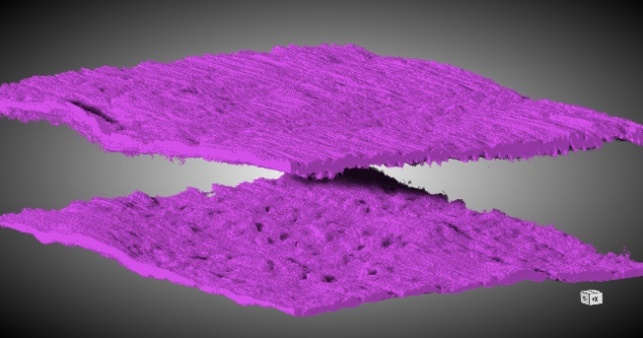

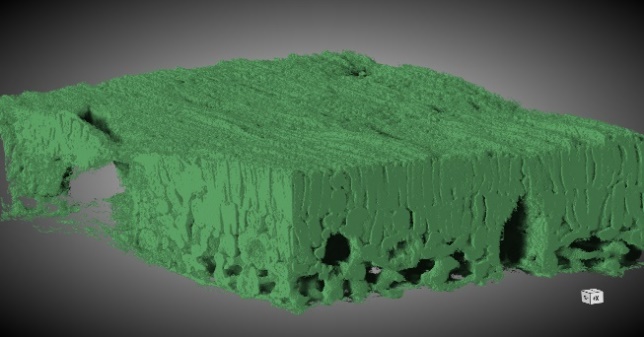

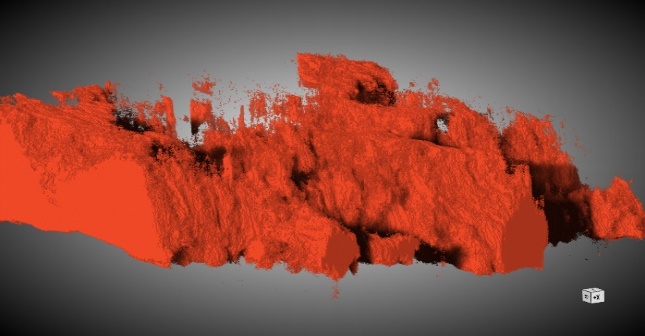

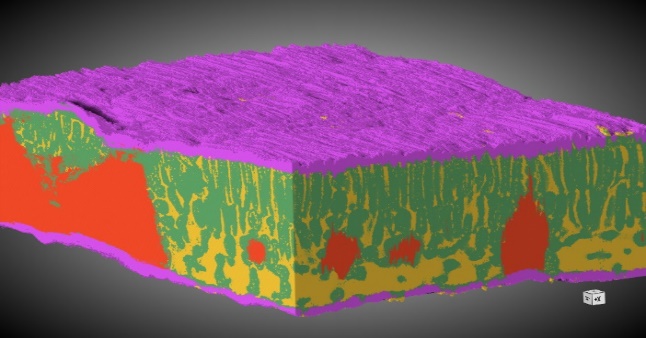


**E**

**F**

**G**

**H**

**A**

**B**

**C**

**D**

**Fig. S3** This figure illustrates steps from image stack preparation to generating auto-segmented stack by machine learning algorithms (A-D) and 3D projection of single tissues to whole leaf construction (E-H); A, grid reconstruction, B, phase reconstruction, C, thresholded (binary) image, D, auto-segmented stack, E, adaxial and abaxial epidermis, F, mesophyll tissue (palisade and spongy cells), G, veins, H, whole leaf 3D construction.


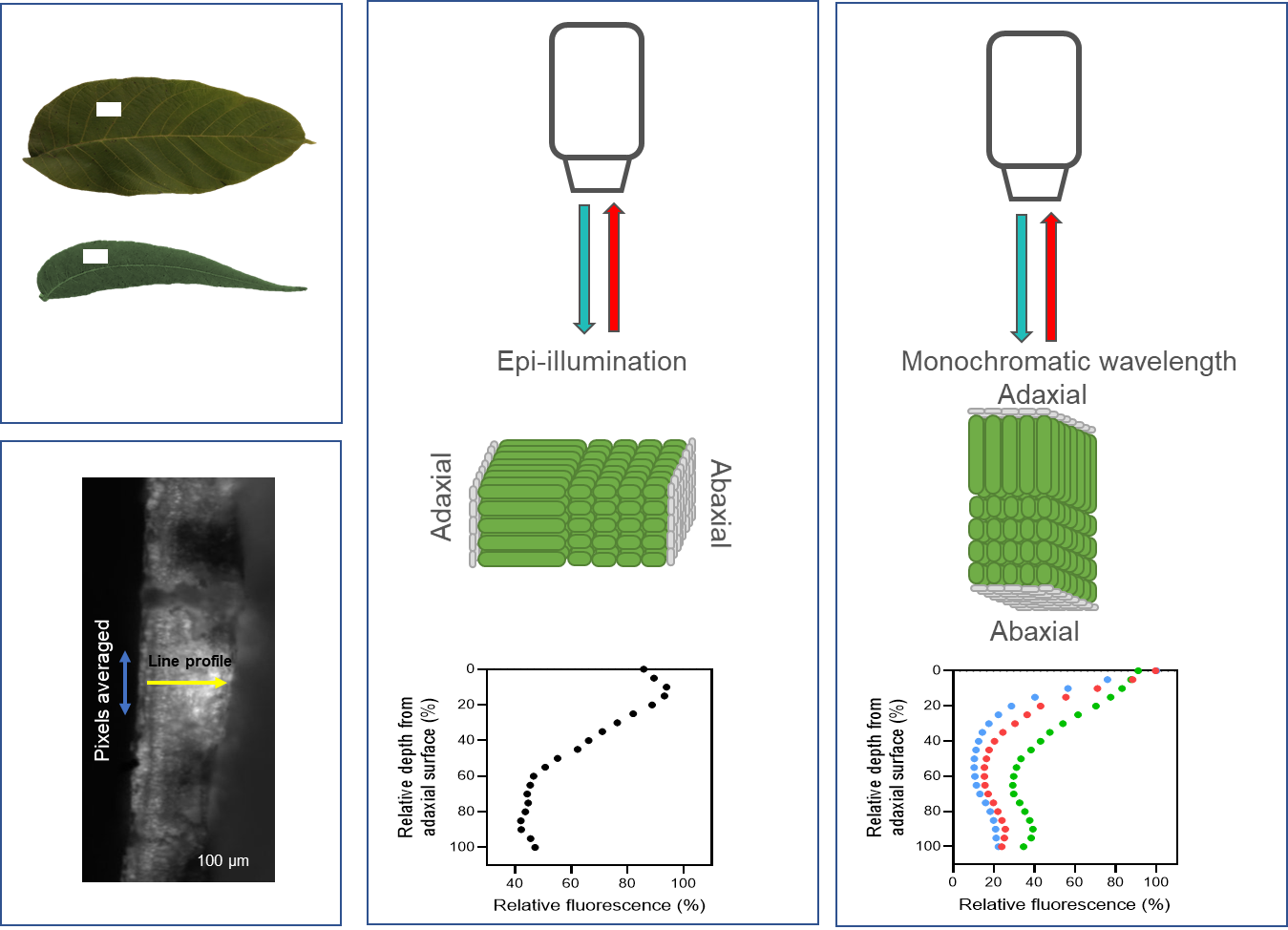


A

B

C

D

**Fig. S4** This figure shows stepwise protocol from sample preparation to plotting light absorption across leaf profile, A, cutting leaf pieces, B, epi-illuminating leaf cross section, C, illuminating leaf adaxial surface by monochromatic wavelengths, D, plotting fluorescence from mesophyll tissue at gray scale.


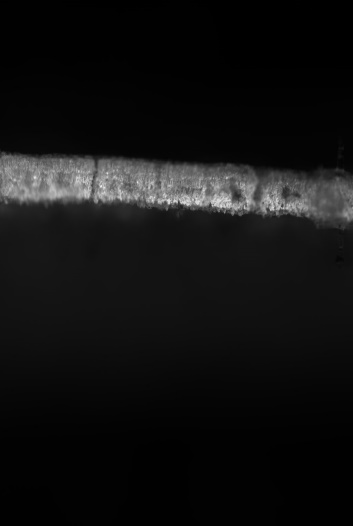


***J. regia***

***J. microcarpa***

**Well-watered**

**Dehydrated**

**Fig. S5** Spatial chlorophyll distribution from original epi-illumination imaging in *J. regia* and *J. microcarpa* under well-watered and dehydrated conditions.

**Adaxial illumination**


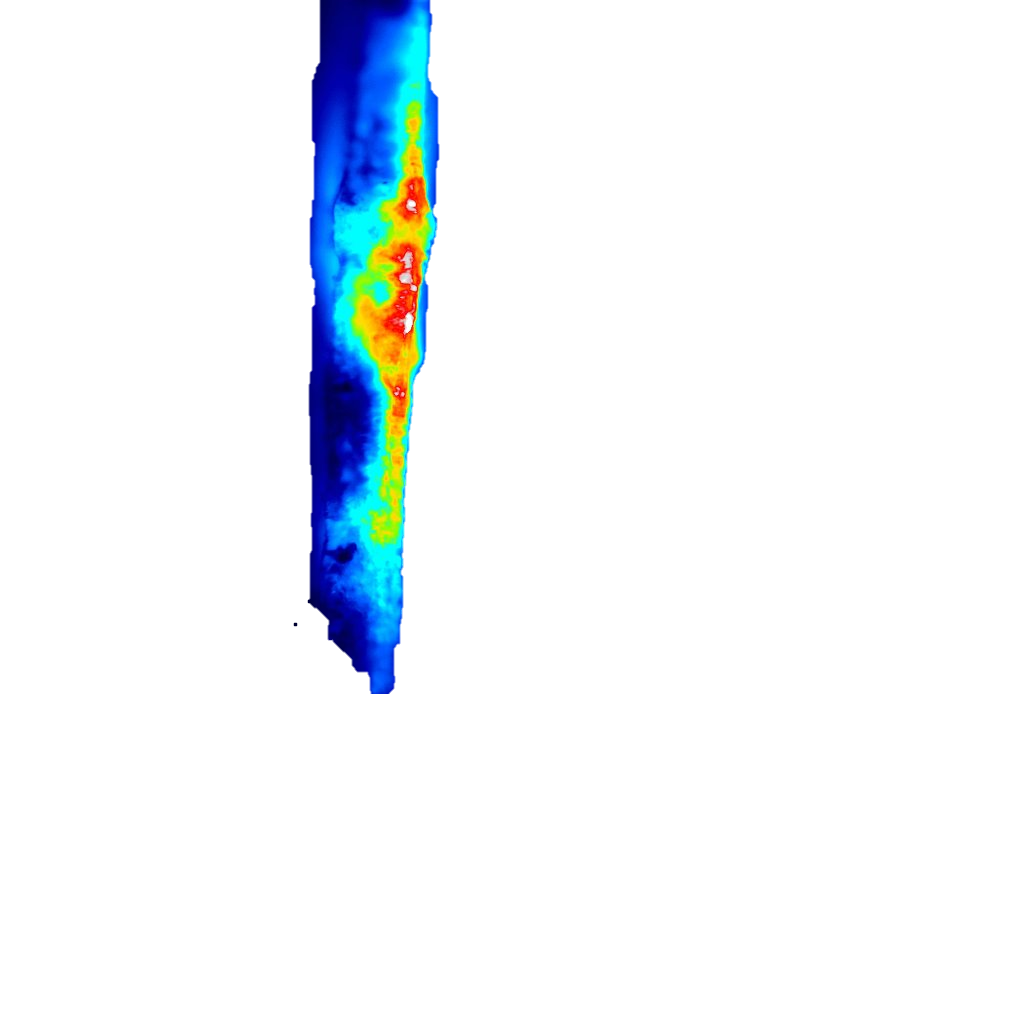

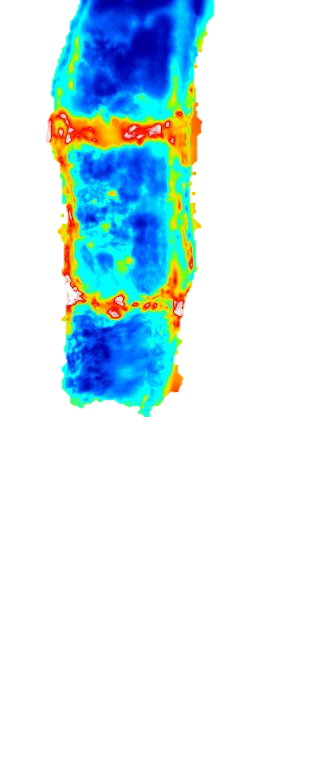

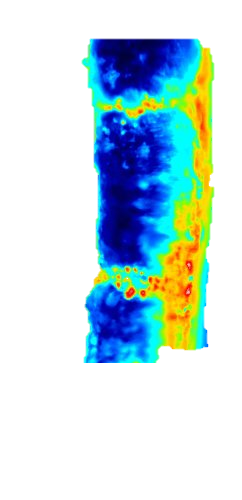

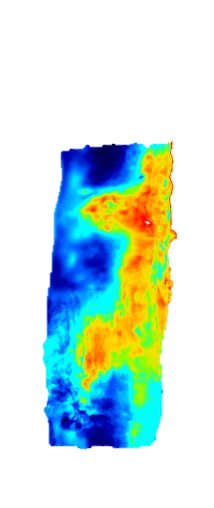


***J. microcarpa***

***J. regia***

**Well-watered**

**Dehydrated**


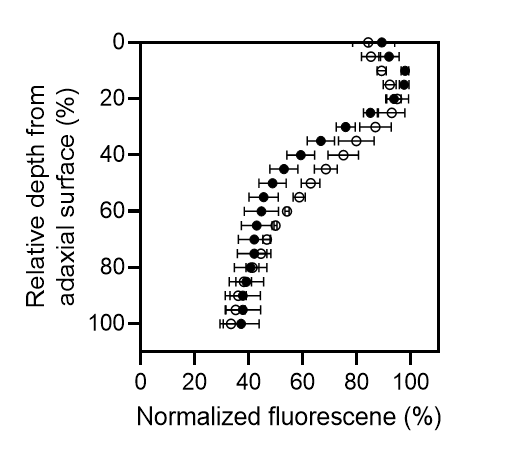

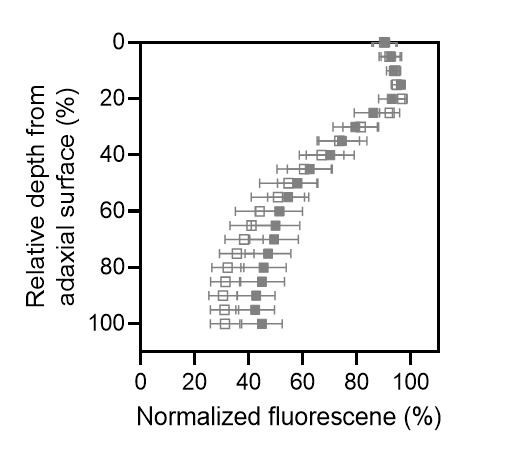


**Fig. S6** Normalized fluorescence near BSEs at adaxial green wavelength illumination in *J. regia* and *J. microcarpa* under well-watered and dehydrated conditions.

**METHODS**

*Mesophyll Conductance (g*_m_*)- Chlorophyll Fluorescence*

To be consistent with the leaf optical measurements (see subsection *Relative Chlorophyll Distribution through the Leaf Profile* below), we chose to use a chlorophyll fluorescence method, the variable *J* method, to estimate *g*_m_. This method is based on calculation of electron transport rate (*J*_flu_) from measurements of chlorophyll fluorescence (Bongi & Loreto, 1989; Harley *et al.,* 1992):

$J_{flu}=\Phi_{\mathrm{PSII}}\times PPFD\times\alpha\times\beta$ (1)

where β (= 0.5 for C_3_ plants) is the fraction of absorbed quanta reaching photosystem II (Bernacchi *et al.,* 2002). The leaf absorbance, α, was estimated to be 85.3% (± 0.2 standard error) by measuring reflectance and transmittance at full wavelength in all individuals using a field spectroradiometer (ASD Fieldspec,ViewSpec Pro, ASD Inc. Boulder, CO, USA). *g*_m_ was given by (Harley *et al.,* 1992):

$g_{m}=A_{n}/\left[ C_{i}-\left( \frac{\Gamma^{*}\left( J_{flu}+8\left( A_{n}+R_{d} \right) \right)}{J_{flu}-4\left( A_{n}+R_{d} \right)} \right) \right]$ (2)

where *R*_d_ is the non-photorespiratory respiration rate in the light, and *Γ** is the chloroplast CO_2_ photocompensation point. *Γ** was assumed to equal the intercellular CO_2_ photocompensation point (*C*_i_*) per Gilbert *et al.* (2012). *R*_d_ (0.73 ± 0.08 µmol m^-2^ s^-1^) and *C*_i_* (38.18 ± 0.47 µmol mol^-1^) were estimated, using the Laisk method (Laisk, 1977 in Gilbert *et al.,* 2012), as the point of intersection of the linear portion of averaged four sets of *A*_n_-*C*_i_ curves obtained at three irradiances (100, 200 and 500 µmol m^-2^ s^-1^) and 13 CO_2_ concentrations (35, 40, 50, 60, 70, 80, 90, 100, 110, 120, 140, 160, and 180 µmol mol^-1^) (Supporting Information Fig. S7).

**Fig. S7** Estimation of CO_2_ compensation point (*Γ**, µmol mol^-1^) and dark respiration (*R*_d_, µmol m^-2^ s^-1^) using the Laisk method (Gilbert et al. 2012, see Materials and Methods for details). Each point represents the mean value for two species in four replicates from our experiment. *A*_n_*-C*_i_ curves were built under three irradiances of 100 (●), 200 (■) and 500 (▲) µmol m^-2^ s^-1^.

**References**

**Bernacchi CJ, Portis AR, Nakano H, von Caemmerer S, Long SP. 2002.** Temperature response of mesophyll conductance; implications for the determination of rubisco enzyme kinetics and for limitations to photosynthesis *in vivo*. *Plant Physiology* **130:** 1992-1998.

**Bongi G, Loreto F. 1989.** Gas-exchange properties of salt-stressed olive (*Olea europea* L.) leaves, *Plant Physiology* **90:** 1408-1416.

**Gilbert ME, Pou A, Zwieniecki MA, Holbrook NM. 2012.** On measuring the response of mesophyll conductance to carbon dioxide with the variable J method. Journal of Experimental Botany **63:** 413-425.

**Harley PC, Loreto F, Di Marco G, Sharkey TD. 1992.** Theoretical considerations when estimating the mesophyll conductance to CO_2_ flux by analysis of the response of photosynthesis to CO_2_. *Plant Physiology* **98:** 1429-1436.
